# The burden of colorectal cancer attributable to diet low in whole grains from 1990 to 2021: a global, regional and national analysis

**DOI:** 10.3389/fnut.2025.1527522

**Published:** 2025-04-09

**Authors:** Yuting Ma, Jinghuai Ni, Pingping Mei, Yan Chen, Xiutian Guo

**Affiliations:** ^1^Shanghai Municipal Hospital of Traditional Chinese Medicine, Shanghai University of Traditional Chinese Medicine, Shanghai, China; ^2^Department of Bone injury of Traditional Chinese Medicine, Affiliated Hospital of Nanjing University of Chinese Medicine, Nanjing, China

**Keywords:** colorectal cancer, diet low in whole grains, death, disability-adjusted life years, global burden of disease colorectal cancer, global burden of disease

## Abstract

**Background:**

Colorectal cancer (CRC) is a major global health issue, with rising incidence and mortality rates. Dietary factors, especially whole grains consumption, are critical in determining CRC risk. Understanding CRC deaths and disability-adjusted life years (DALYs) related to low whole grains diets is important for prevention. The purpose of the study is to investigate temporal and geographic trends in CRC deaths and DALYs attributable to diet low in whole grains at the global, regional, and national levels from 1990 to 2021.

**Methods:**

The data on CRC burden attributable to diet low in whole grains from 1990 to 2021 were extracted from the Global Burden of Diseases (GBD) 2021 database. We described the CRC burden attributable to diet low in whole grains across various years, genders, age groups (5-year age groups from 25 to 94 years and 95+ years), different Socio-demographic Index (SDI) regions and countries. To illustrate the temporal trends in the burden of CRC, we calculated the estimated annual percentage change (EAPC) from 1990 to 2021.

**Results:**

From 1990 to 2021, the global number of CRC deaths attributable to diet low in whole grains increased from 101,813 (95% UI: 42,588 to 151,170) to 186,257 (95% UI: 76,127 to 284,803), representing a 82.94% growth. Similarly, the number of DALYs increased from 2,540,867 (95% UI: 1,050,794 to 3,754,416) to 4,327,219 (95% UI: 1,754,865 to 6,578,232), representing a 70.30% growth. However, both the age-standardized mortality rate (ASMR) and age-standardized DALY rate (ASDR) exhibited a decline, with an EAPC of −0.82 (95% CI: −0.85 to −0.78) and − 0.84 (95% CI: −0.87 to −0.81), respectively. The disease burden is heavier in high SDI and high-middle SDI regions. However, between 1990 and 2021, the only region where both ASMR and ASDR increased was low-middle SDI, while in all other regions, they showed a declining trend. In 2021, East Asia had the highest number of CRC deaths and DALYs attributable to diet low in whole grains at the regional level, followed by Western Europe and High-income North America. Additionally, the burden is greater among males and the elderly. Between 1990 and 2021, the number of CRC deaths attributable to diet low in whole grains rose by 102.13% among males and by 63.20% among females. Generally, both the global age-specific mortality rate and the DALYs rate tend to increase with age. SDI demonstrates a nonlinear “S”-shaped correlation with both ASMR and ASDR of CRC attributable to diet low in whole grains. In 2021, the EAPC in ASMR of CRC attributable to diet low in whole grains was negatively associated with SDI (*R* = −0.402, *p* < 0.001), reaching the highest EAPC at approximately SDI of 0.51 and the lowest at 0.85. Similarly, the correlation between EAPC in ASDR and SDI in 2021 exhibited a similar pattern.

**Conclusion:**

Despite a decline in the ASMR and ASDR of CRC attributable to diet low in whole grains from 1990 to 2021 globally, the absolute number of cases continues to increase, with a particularly notable burden observed in High-middle and High SDI regions, as well as among males and the elderly population. It is imperative to intensify efforts in CRC prevention and health education, specifically targeting these high-risk groups to raise public awareness and consumption of whole grains. Furthermore, screening initiatives should be intensified among these demographics to address the elevated risk of CRC mortality due to insufficient whole grains consumption.

## 1 Introduction

Colorectal cancer (CRC) refers to cancer that occur in the colon or rectum, which ranks as the third most commonly diagnosed cancer and holds the second position in terms of cancer-related death globally. Its incidence and mortality account for 9.6 and 9.3% of new cancer cases and deaths worldwide, respectively (1). According to the predictions of human disease progression, the global burden of CRC is expected to increase to 3.2 million new cases and 1.6 million deaths by 2040 (2).

Today, highly developed countries remain high-risk areas, such as Australia, New Zealand, Canada and others. The incidence of CRC is about three fold higher in high and very high human development index (HDI) countries compared to low and medium HDI countries (3). The mortality rate of early-onset colorectal cancer (below 50 years of age) is also on the rise. In the 1990s, CRC ranked as the fourth leading cause of cancer-related death among both men and women under 50 in the United States, however, it has since risen to become the leading cause of cancer death in men and the second leading cause in women within this age group (4).

In 2022, China saw 517,000 new cases of colorectal cancer, accounting for 10% of all newly diagnosed malignant tumors and ranking second in the incidence spectrum (5). Obesity, physical inactivity, poor diets, alcohol drinking and smoking are the causes of the high incidence of CRC in highly developed countries (6). With the Westernization of lifestyles, the incidence and mortality of CRC are rising rapidly and the burden is increasing in many less-developed countries, especially in Eastern Europe, Asia and South America (3, 7). CRC, as a malignant tumor that poses a serious threat to human health, necessitates the formulation and implementation of prevention strategies to alleviate the disease burden at both the societal and individual levels.

CRC is a multifactorial disease. Its onset involves the complex interplay of various factors such as genetics, environment, dietary habit and lifestyle (8, 9). Numerous studies have demonstrated that diets low in whole grains is associated with an increased risk of CRC (10–12). Whole grains, such as brown rice, oats, and whole wheat bread, retain more nutrients compared to refined grains (like white rice and white flour products), including B vitamins, magnesium, iron, and dietary fiber. The dietary fiber in whole grains can promote intestinal motility, increase stool volume, dilute carcinogens, affect bile acid metabolism, and enhance the production of short-chain fatty acids, thereby reducing the risk of CRC (13, 14). Polyphenols, as another pivotal functional compound in whole grains, can exert an inhibitory effect on colorectal cancer by influencing the colonic microbiota (15) and the inflammatory process (16). A prospective study has found that for additional 30 g/day of whole grains intake, the risk of CRC can be reduced by approximately 17% (17).

However, the epidemiological research studies of CRC burden attributable to diet low in whole grains at the global, regional, and national levels remains unclear. To fill this critical gap in the literature, our objective was to investigate the global, regional, national burden of CRC deaths and disability-adjusted life-years (DALYs) attributable to diet low in whole grains between 1990 and 2021.

## 2 Materials and methods

### 2.1 Data source

All data were from the GBD 2021 through their official website at https://GHDx.healthdata.org/GBD-2021/GBD-results-tool. The GBD is a publicly accessible database, that provides estimates of 371 diseases and injuries burden in 204 countries and regions from 1990 to 2021, including incidence, prevalence, mortality, Disability-Adjusted Life Years (DALYs), Years Lived with Disability (YLDs), Years of Life Lost (YLLs) and healthy life expectancy (HALE) (18). We obtained the CRC deaths, DALYs, age-standardized mortality rates (ASMR), and age-standardized DALY rates (ASDR) due to a diet low in whole grains from GBD 2021. Based on the extracted data, we described the CRC burden attributable to diet low in whole grains across various years, genders, age groups (5-year age groups from 25 to 94 years and 95+ years), different SDI regions and countries.

### 2.2 Definitions

Whole grains intake is defined as the average daily consumption (in grams per day) of whole grains and their products, which encompass breakfast cereals, breads, rice, biscuits, muffins, tortillas, pancakes, pasta, and other related sources. A diet low in whole grains is defined as an average daily consumption is less than 140-160 g/d (19). In GBD 2021, CRC is defined as C18–C21.9, D01.0–D01.3, D12–D12.9, D37.3–D37.5. As a comprehensive indicator of socio-demographic development, the Socio-Demographic Index (SDI) is derived from an extensive assessment that includes the total fertility rate of females younger than 25 years old, average education level of those aged 15 years and older, per capita income distribution, ranged from 0 to 1 (18). It exhibits a strong correlation with indicators related to healthy life years and disease burden, making it a valuable tool for evaluating the health development status and disease burden across countries and regions. The SDI values divide all countries and regions into five levels: low SDI, low-middle SDI, middle SDI, high-middle SDI, and high SDI. DALY is an indicator used to measure the burden of disease. It quantifies the comprehensive loss to population health caused by illness, disability, and premature death by combining the years of premature mortality and years lived with disability (18, 20).

### 2.3 Statistical analysis

The burden of CRC attributable to diet low in whole grains was quantified based on the number of deaths, DALYs, ASMR, and ASDR. ASR a classical epidemiological method used to standardize the incidence or mortality rate of a certain health event across different countries or regions, as well as over various time periods. This method allows for rate data from different regions and populations to be compared in a comparable manner. ASR is calculated as follows:


$$ASR=\frac{\sum_{i=1}^{A}a_{i}w_{i}}{\sum_{i=1}^{A}w_{i}}\times 100,100\text{,}$$

where *a_i_* is the age-specific rate (*i* denotes the *i*th age class) and *w_i_* is the population count (or weight) corresponding to the specific age group *i* in the selected standard population. All rates are expressed as per 100,000 people. Additionally, estimated Annual Percentage Change (EAPC) is often used to analyze the trends of indicators such as ASMR or ASDR from 1990 to 2021, which is calculated as EAPC = 100(exp(*β*)-1) (21). By calculating EAPC, we can know the annual percentage change of ASR over a period of time, thereby judging the trend of disease burden in different countries and regions. For instance, if both the EAPC and its 95% confidence interval are>0, it signifies an annual increase in ASR. Conversely, if both the EAPC and its 95% confidence interval are<0, which indicates a decrease in ASR. What’s more, the ASR would be regarded as stable if the 95% CI contained 0. Finally, to identify the factors influencing EAPC, we conducted a Pearson correlation analysis to examine the correlation between ASMR or ASDR in 1990, SDI in 2021 and EAPC at the national level. All statistical analyses and data visualization were performed using R software (version 4.4.1) (22).

## 3 Results

### 3.1 Global burden of CRC attributable to diet low in whole grains from 1990 to 2019

From 1990 to 2021, the global number of CRC deaths attributable to diet low in whole grains increased from 101,813 (95% UI: 42,588 to 151,170) to 186,257 (95% UI: 76,127 to 284,803), representing a 82.94% growth. Similarly, the number of DALYs increased from 2,540,867 (95% UI: 1,050,794 to 3,754,416) to 4,327,219 (95% UI: 1,754,865 to 6,578,232), representing a 70.30% growth. However, the ASMR decreased from 2.79 per 100,000 population (95% UI: 1.17 to 4.15) to 2.21 per 100,000 population (95% UI: 0.91 to 3.38), with an EAPC of −0.82 (95% CI: −0.85 to −0.78). Similarly, the ASDR declined from 63.47 per 100,000 people (95% UI: 26.35 to 93.84) to 50.19 per 100,000 population (95% UI: 20.37 to 76.30), with an EAPC of −0.84 (95% CI: −0.87 to −0.81) (Table 1).

**Table 1:** Burden of CRC attributable to diet low in whole grains in Global, SDI regions, and GBD regions.

Characteristics	1990				2021				EAPC(1990–2021)	
	Deaths Cases	ASMR (/100 k)	DALYs	ASDR (/100 k)	Deaths Cases	ASMR (/100 k)	DALYs	ASDR (/100 k)	ASMR	ASDR
	No. (95% UI)	No. (95% UI)	No. (95% UI)	No. (95% UI)	No. (95% UI)	No. (95% UI)	No. (95% UI)	No. (95% UI)	No. (95% CI)	No. (95% CI)
Global	101,813 (42,588 to 151,170)	2.79 (1.17 to 4.15)	2,540,867 (1,050,794 to 3,754,416)	63.47 (26.35 to 93.84)	186,257 (76,127 to 284,803)	2.21 (0.91 to 3.38)	4,327,219 (1,754,865 to 6,578,232)	50.19 (20.37 to 76.30)	−0.82 (−0.85 to −0.78)	−0.84 (−0.87 to −0.81)
Sex										
Male	51,622 (21,646 to 767,288)	3.19 (1.35 to 4.78)	1,349,763 (563,930 to 2,011,923)	72.49 (30.36 to 107.77)	104,344 (42,110 to 159,294)	2.75 (1.11 to 4.20)	2,527,992 (1,020,944 to 3,876,411)	62.39 (25.20 to 95.64)	−0.5 (−0.53 to −0.48)	−0.52 (−0.55 to −0.5)
Female	50,191 (20,942 to 74,790)	2.47 (1.04 to 3.69)	1,191,104 (486,865 to 1,765,199)	55.85 (22.91 to 82.83)	81,912 (34,184 to 123,364)	1.76 (0.74 to 2.66)	1,799,227 (736,335 to 2,717,497)	39.36 (16.10 to 59.45)	−1.21 (−1.26 to −1.16)	−1.27 (−1.32 to −1.21)
SDI region										
High SDI	43,043 (18,345 to 64,229)	3.87 (1.65 to 5.77)	941,428 (398,133 to 1,402,354)	86.30 (36.51 to 128.53)	60,473 (25,200 to 92,184)	2.70 (1.12 to 4.10)	1,205,653 (500,387 to 1,815,640)	60.69 (25.12 to 91.16)	−1.25 (−1.28 to −1.21)	−1.21 (−1.24 to −1.17)
High-middle SDI	31,690 (13,146 to 46,873)	3.36 (1.40 to 4.98)	808,498 (332,629 to 1,194,207)	80.20 (33.06 to 118.50)	56,721 (22,911 to 86,095)	2.89 (1.17 to 4.38)	1,298,820 (519,592 to 1,965,736)	66.60 (26.65 to 100.77)	−0.55 (−0.62 to −0.49)	−0.71 (−0.77 to −0.65)
Middle SDI	18,435 (7,398 to 28,070)	1.89 (0.77 to 2.87)	537,904 (215,134 to 819,573)	47.61 (19.10 to 72.49)	47,738 (19,197 to 73,112)	1.85 (0.74 to 2.83)	1,230,182 (494,733 to 1,879,459)	44.72 (17.98 to 68.32)	−0.13 (−0.17 to −0.09)	−0.25 (−0.3 to −0.2)
Low-middle SDI	5,748 (2,417 to 8,914)	0.98 (0.41 to 1.52)	170,154 (70,941 to 262,876)	25.28 (10.62 to 39.13)	15,442 (6,372 to 22,974)	1.11 (0.46 to 1.65)	430,073 (176,445 to 643,410)	28.05 (11.53 to 41.95)	0.45 (0.42 to 0.48)	0.36 (0.34 to 0.39)
Low SDI	2,751 (1,161 to 4,291)	1.29 (0.54 to 2.02)	79,389 (33,441 to 123,673)	32.35 (13.68 to 50.39)	5,640 (2,327 to 8,440)	1.23 (0.51 to 1.84)	157,203 (64,962 to 233,832)	28.75 (11.87 to 42.88)	−0.24 (−0.35 to −0.14)	−0.51 (−0.61 to −0.41)
GBD regions										
Oceania	30 (12 to 48)	1.17 (0.47 to 1.81)	933 (373 to 1,455)	28.41 (11.49 to 44.39)	70 (28 to 107)	1.03 (0.41 to 1.56)	2,114 (840 to 3,231)	25.10 (9.99 to 38.13)	−0.37 (−0.47 to −0.27)	−0.39 (−0.48 to −0.3)
Southeast Asia	3,950 (1,640 to 6,043)	1.62 (0.68 to 2.49)	115,594 (47,680 to 177,860)	41.08 (17.03 to 63.08)	12,528 (5,042 to 18,993)	2.01 (0.81 to 3.04)	340,389 (136,102 to 511,101)	49.19 (19.70 to 74.27)	0.67 (0.61 to 0.74)	0.56 (0.5 to 0.62)
East Asia	22,088 (8,771 to 34,016)	2.78 (1.10 to 4.27)	646,779 (257,106 to 994,560)	69.28 (27.54 to 106.65)	52,283 (20,976 to 83,298)	2.50 (1.00 to 3.97)	1,295,744 (524,660 to 2,051,943)	60.18 (24.33 to 95.13)	−0.42 (−0.47 to −0.36)	−0.54 (−0.6 to −0.47)
Central Europe	5,915 (2,478 to 8,741)	4.06 (1.70 to 6.00)	141,072 (58,975 to 208,942)	94.12 (39.32 to 139.42)	9,495 (3,942 to 14,240)	4.13 (1.71 to 6.20)	199,028 (82,275 to 297,982)	92.49 (38.15 to 138.76)	−0.05 (−0.17 to 0.07)	−0.15 (−0.27 to −0.03)
Central Asia	908 (379 to 1,365)	1.95 (0.82 to 2.94)	25,987 (10,919 to 39,252)	52.45 (22.00 to 79.19)	1,163 (481 to 1,784)	1.49 (0.62 to 2.29)	31,919 (13,174 to 48,964)	36.92 (15.25 to 56.58)	−0.48 (−0.63 to-0.34)	−0.86 (−0.97 to −0.74)
Eastern Europe	9,453 (3,920 to 14,006)	3.41 (1.41 to 5.04)	240,975 (99,959 to 357,627)	85.55 (35.48 to 126.94)	12,007 (5,039 to 17,766)	3.36 (1.41 to 4.98)	273,010 (114,999 to 401,921)	78.90 (33.27 to 116.43)	−0.24 (−0.35 to −0.13)	−0.52 (−0.65 to −0.39)
Australasia	1,009 (421 to 1,533)	4.35 (1.82 to 6.61)	23,317 (9,745 to 35,119)	100.80 (42.14 to 151.66)	1,479 (602 to 2,259)	2.61 (1.08 to 4.00)	29,863 (12,591 to 45,527)	58.54 (25.00 to 88.69)	−1.85 (−1.94 to −1.76)	−1.99 (−2.09 to −1.89)
High-income Asia Pacific	5,843 (2,461 to 8,831)	3.03 (1.28 to 4.59)	142,754 (59,701 to 214,811)	70.50 (29.53 to 106.10)	13,974 (5,931 to 21,445)	2.60 (1.09 to 3.96)	250,021 (105,027 to 380,218)	57.34 (23.88 to 86.30)	−0.52 (−0.58 to −0.47)	−0.71 (−0.77 to −0.65)
Southern Latin America	1,697 (710 to 2,525)	3.81 (1.60 to 5.67)	38,793 (16,123 to 57,863)	83.94 (34.95 to 125.16)	3,020 (1,248 to 4,598)	3.39 (1.40 to 5.17)	65,167 (26,905 to 99,425)	75.90 (31.31 to 115.88)	−0.14 (−0.3 to 0.02)	−0.09 (−0.23 to 0.05)
Western Europe	24,785 (10,511 to 36,960)	4.18 (1.77 to 6.23)	510,578 (215,360 to 765,413)	90.32 (38.19 to 135.28)	28,985 (11,875 to 43,653)	2.79 (1.16 to 4.19)	538,523 (222,514 to 804,551)	60.30 (24.79 to 89.42)	−1.34 (−1.39 to −1.29)	−1.34 (−1.4 to −1.29)
High-income North America	13,194 (5,633 to 19,617)	3.67 (1.57 to 5.45)	286,863 (121,592 to 423,918)	83.39 (35.33 to 123.23)	15,390 (6,564 to 23,033)	2.32 (0.99 to 3.46)	342,013 (144,494 to 508,975)	56.56 (23.83 to 84.12)	−1.61 (−1.68 to −1.55)	−1.34 (−1.39 to −1.28)
Caribbean	637 (272 to 960)	2.57 (1.09 to 3.86)	15,190 (6,477 to 22,950)	57.97 (24.72 to 87.64)	1,426 (562 to 2,167)	2.64 (1.04 to 4.00)	32,389 (12,894 to 49,039)	60.31 (24.05 to 91.36)	0.21 (0.17 to 0.24)	0.26 (0.22 to 0.3)
Andean Latin America	311 (133 to 475)	1.61 (0.69 to 2.46)	7,809 (3,322 to 11,900)	36.67 (15.64 to 55.90)	1,024 (392 to 1,605)	1.77 (0.68 to 2.78)	23,979 (9,183 to 37,638)	40.00 (15.30 to 62.81)	0.38 (0.27 to 0.48)	0.3 (0.19 to 0.41)
Tropical Latin America	1,427 (590 to 2,143)	1.69 (0.70 to 2.55)	38,033 (15,692 to 56,901)	39.42 (16.28 to 59.13)	5,029 (2,144 to 7,573)	1.98 (0.84 to 2.98)	126,986 (54,321 to 191,291)	48.70 (20.82 to 73.35)	0.53 (0.45 to 0.62)	0.64 (0.55 to 0.74)
Central Latin America	926 (380 to 1,366)	1.20 (0.49 to 1.77)	24,187 (9,946 to 35,390)	27.34 (11.24 to 40.18)	3,795 (1,513 to 5,783)	1.54 (0.61 to 2.34)	97,676 (39,089 to 148,606)	38.15 (15.26 to 58.09)	0.84 (0.76 to 0.92)	1.09 (1.01 to 1.17)
South Asia	4,280 (1,859 to 6,564)	0.76 (0.33 to 1.17)	131,068 (56,845 to 200,092)	20.12 (8.75 to 30.74)	11,356 (4,736 to 17,077)	0.79 (0.33 to 1.18)	320,918 (133,327 to 481,047)	20.32 (8.45 to 30.54)	0.01 (−0.09 to 0.11)	−0.1 (−0.2 to 0.00)
North Africa and Middle East	2,638 (1,070 to 3,947)	1.69 (0.69 to 2.53)	75,303 (31,144 to 112,922)	41.27 (16.87 to 62.03)	7,010 (2,867 to 10,717)	1.68 (0.69 to 2.57)	188,025 (75,808 to 285,507)	38.97 (15.81 to 59.47)	0.2 (0.05 to 0.35)	−0.02 (−0.15 to 0.11)
Central Sub-Saharan Africa	256 (109 to 397)	1.28 (0.55 to 1.99)	7,454 (3,138 to 11,527)	30.78 (13.03 to 47.79)	645 (261 to 1,088)	1.31 (0.54 to 2.23)	18,961 (7,606 to 31,747)	31.16 (12.64 to 52.56)	0.11 (−0.02 to 0.23)	0.07 (−0.05 to 0.18)
Western Sub-Saharan Africa	753 (307 to 1,152)	0.95 (0.39 to 1.45)	19,755 (8,019 to 30,293)	21.74 (8.89 to 33.34)	1,833 (788 to 2,757)	1.07 (0.46 to 1.60)	48,528 (20,549 to 73,776)	23.49 (10.12 to 35.34)	0.56 (0.49 to 0.62)	0.4 (0.34 to 0.45)
Eastern Sub-Saharan Africa	1,365 (575 to 2,131)	1.95 (0.82 to 3.04)	38,964 (16,326 to 60,927)	48.22 (20.27 to 75.34)	2,774 (1,189 to 4,140)	1.89 (0.82 to 2.82)	75,703 (31,963 to 112,169)	42.13 (18.01 to 62.87)	−0.24 (−0.33 to −0.15)	−0.62 (−0.72 to −0.52)
Southern Sub-Saharan Africa	347 (146 to 543)	1.38 (0.58 to 2.15)	9,460 (3,979 to 14,869)	32.51 (13.66 to 50.92)	969 (409 to 1,476)	1.81 (0.76 to 2.74)	26,263 (11,059 to 39,903)	42.77 (18.08 to 65.07)	1.01 (0.74 to 1.28)	1.11 (0.82 to 1.39)

SDI, Socio-demographic Index; GBD, Global burden of disease study; ASMR, Age-standardized mortality rate; DALYs, Disability-adjusted life years; ASDR, Age-standardized DALY rate; EAPC, Estimated annual percentage changes; UI, Uncertainty interval; CI, confidence interval.

**Figure 1:**
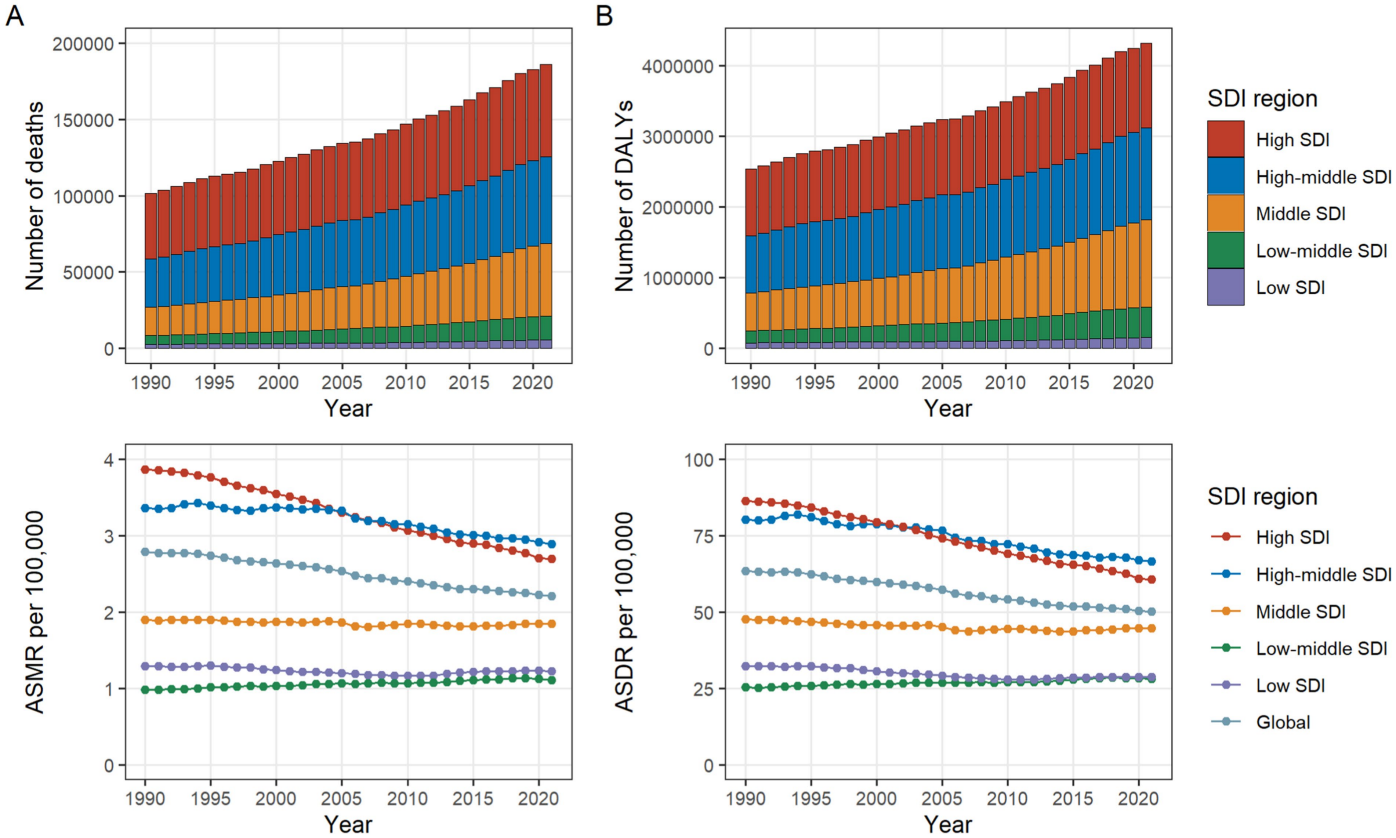
Number and rate of colorectal cancer deaths **(A)** and DALYs **(B)** attributable to diet low in whole grains from 1990 to 2019 by SDI region. The bars represent the number of colorectal cancer deaths **(A)** and DALYs **(B)** attributable to diet low in whole grains from 1990 to 2019 in the SDI regions. The line represents ASMR **(A)** and ASDR **(B)** (per 100,000) attributable to diet low in whole grains from 1990 to 2019 in the global and SDI regions. ASMR, Age-standardized mortality rate; ASDR, Age-standardized DALY rate; DALYs, Disability-adjusted life years; SDI, Socio-demographic index.

At the SDI regional level, the number of CRC deaths attributable to diet low in whole grains was highest in high SDI regions and lowest in low SDI regions in both 1990 and 2021. In 1990, the number of CRC DALYs attributable to diet low in whole grains was highest in high SDI regions, at 941,428 (95% UI: 398,133 to 1,402,354). While in 2021, the highest number of DALYs was observed in high-middle SDI regions, at 1,298,820 (95% UI: 519,592 to 1,965,736). At the same time, high-middle SDI regions had the greatest ASMR [2.89 (95% UI: 1.17 to 4.38)] and ASDR [66.60 (95% UI: 26.65 to 100.77)] per 100,000, respectively. Between 1990 and 2021, the only region where both ASMR and ASDR increased was Low-middle SDI, while they declined in all other regions (Table 1; Figures 1A,B).

At the regional level, East Asia had the highest number of CRC deaths [52,283 (95% UI: 20,976 to 83,298)] and DALYs [1,295,744 (95% UI: 524,660 to 2,051,943)] attributable to diet low in whole grains in 2021, followed by Western Europe and High-income North America. Also in 2021, South Asia had the lowest ASMR [0.79 (95% UI: 0.33 to 1.18)] and ASDR [20.32 (95% UI: 8.45 to 30.54)], while Central Europe had the highest ASMR [4.13 (95% UI: 1.71 to 6.20)] and ASDR [92.49 (95% UI: 38.15 to 138.76)]. Australasia showed the largest decrease in the ASMR and ASDR of CRC attributable to diet low in whole grains, with an EAPC of −1.85 (95% CI: −1.94 to −1.76) in ASMR and an EAPC of −1.99 (95% CI: −2.09 to −1.89) in ASDR. While, Southern Sub-Saharan Africa showed the largest increase with an EAPC of 1.01 (95% CI: 0.74 to 1.28) in ASMR and an EAPC of 1.11 (95% CI: 0.82 to 1.39) in ASDR (Table 1).

**Figure 2:**
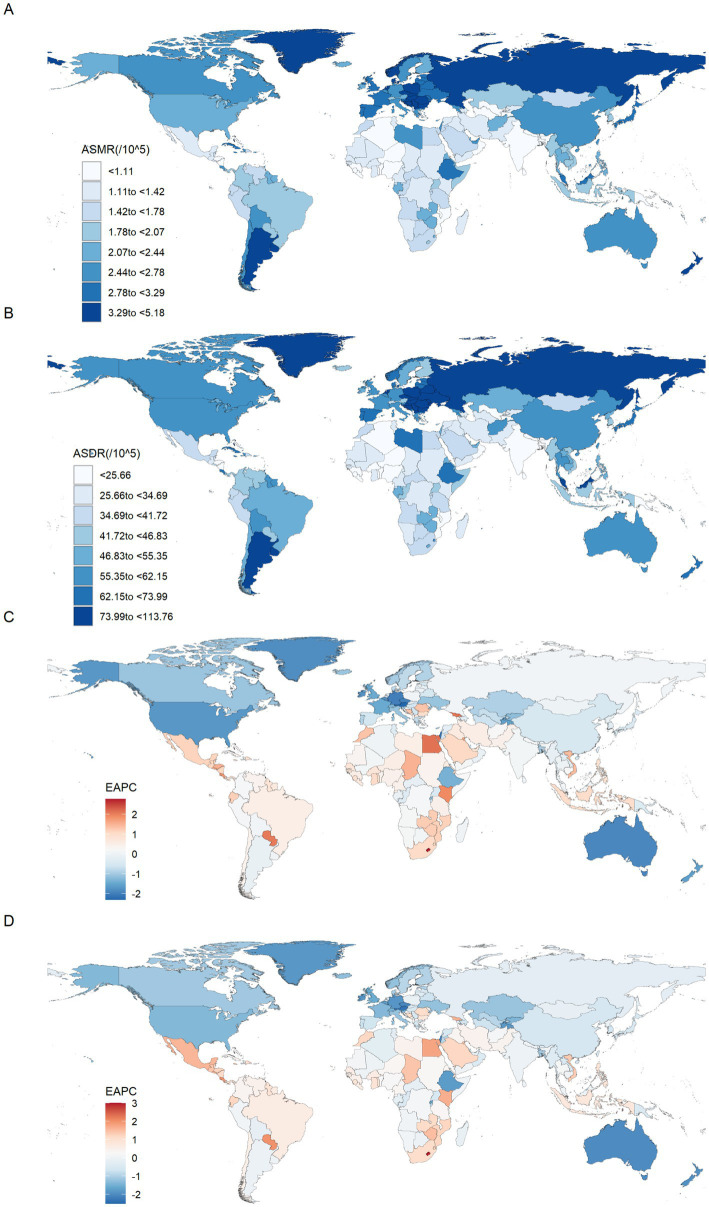
The spatial distribution of the colorectal cancer ASMR **(A)** and ASDR **(B)** attributable to diet low in whole grains in 2021, and the EAPC in colorectal cancer ASMR **(C)** and ASDR **(D)** attributable to diet low in whole grains. ASMR, Age-standardized mortality rate; ASDR, Age-standardized DALY rate; EAPC, Estimated annual percentage changes.

At the national level, China had the highest number of CRC deaths [49,991 (95% UI: 20,100 to 79,929)] and DALYs [1,241,928 (95% UI: 503,165 to 1,978,508)] attributable to diet low in whole grains in 2021. The number of deaths increased by 134.37% compared to 1990, and the DALYs increased by 98.73% compared to 1990. The United States of America followed closely, with 13,474 (95% UI: 5,747 to 20,241) and 303,637 (95% UI: 129,374 to 454,027), respectively. In contrast, Niue, Tokelau, Nauru, Tuvalu and Cook Islands showed the lowest level. Also in 2021, the highest ASMR was found in Uruguay [5.18 (95% UI: 2.09 to 7.73)], followed by Hungary, Bulgaria, Monaco, Slovakia, Poland, Croatia, Greenland and Barbados, with an ASMR of over 4 per 100,000 population. Meanwhile, Hungary had the highest ASDR, at 113.76 (95% UI: 47.61 to 173.37) in 2021, followed by Bulgaria, Uruguay, Slovakia, Greenland and Monaco, with an ASDR of over 100 per 100,000 population (Supplementary Table S1; Figures 2A,B). Notably, Lesotho had the highest increases in ASMR and ASDR from 1990 to 2021, with EAPCs of 2.77 (95% CI: 2.37 to 3.17) and 3.00 (95% CI: 2.56 to 3.44), followed by Cabo Verde. In contrast, Austria experienced the fastest decline in ASMR from 1990 to 2021, with an EAPC of −2.32 (95% CI: −2.37 to −2.27), followed by Israel, Maldives, Singapore and Germany. Maldives experienced the fastest decline in ASDR, with an EAPC of −2.52 (95% CI: −2.65 to −2.38), followed by Austria, Singapore, Israel and Luxembourg (Supplementary Table S1; Figures 2C,D).

### 3.2 Global burden of CRC attributable to diet low in whole grains by age and sex

**Figure 3:**
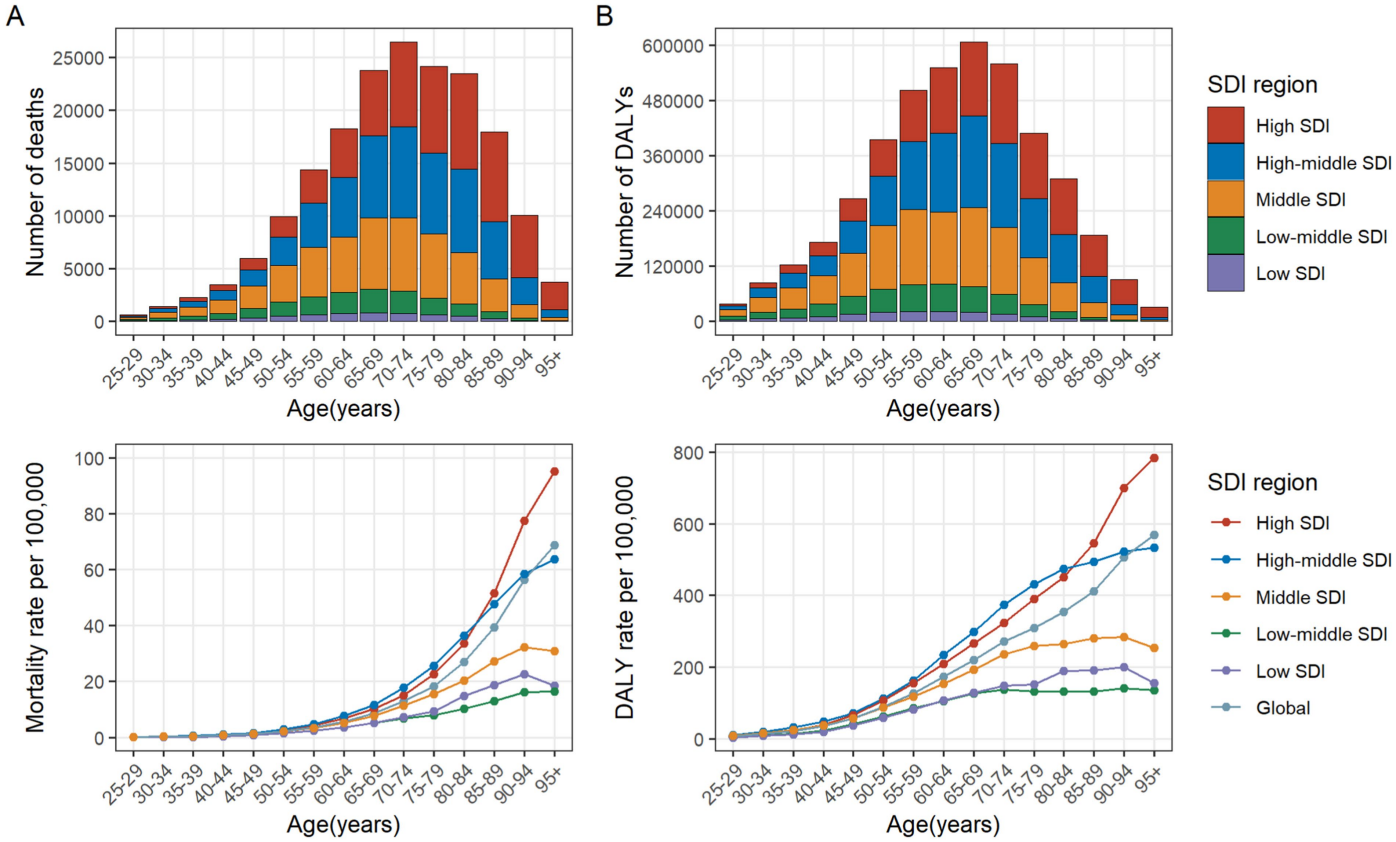
Number and rate of colorectal cancer deaths **(A)** and DALYs **(B)** attributable to diet low in whole grains by age group and SDI region in 2021. The bars represent the number of colorectal cancer deaths **(A)** and DALYs **(B)** attributable to diet low in whole grains among different age groups in the SDI regions in 2021. The line represents the rates of mortality **(A)** and DALYs **(B)** of colorectal cancer due to diet low in whole grains among different age groups in the global and SDI regions in 2021. DALYs, Disability-adjusted life years; SDI, Socio-demographic index.

**Figure 4:**
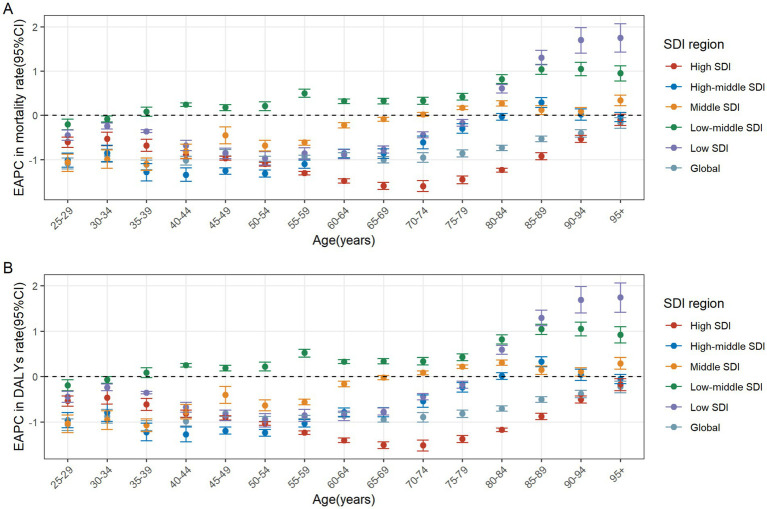
The age distribution of the trends in colorectal cancer mortality rate **(A)** and DALYs rate **(B)** attributable to diet low in whole grains from 1990 to 2021 by SDI region. DALYs, Disability-adjusted life years; EAPC, Estimated annual percentage changes; CI, confidence interval; SDI, Socio-demographic index.

In 2021, the number of CRC deaths attributable to diet low in whole grains globally was predominantly concentrated in the 70–74 age group, while DALYs were mainly concentrated in the 65–69 age group. In the case of individuals under the age of 60, the greatest numbers of deaths and DALYs are observed in the middle SDI regions. For those between the ages of 60 and 75, the greatest numbers are seen in high-middle SDI regions. Finally, for individuals aged 75 and above, the greatest numbers are observed in high SDI regions. In general, the global age-specific mortality rate and the DALYs rate increase with age. However, in the low and middle SDI regions, the age-specific mortality and DALY rates exhibit a decline at ages above 90. In the low-middle SDI region, the age-specific DALYs rate demonstrates a fluctuating decline when age reaches 70 and above (Figures 3A,B). From 1990 to 2021, the age-specific mortality rate and DALYs rate for all age groups globally exhibited a downward trend, with the largest decline observed in the 35–39 age group. In the high SDI regions, the age-specific mortality rate for the 25–94 age group exhibited a decline, whereas the rate for those over 95 remained stable. The age-specific DALYs rate exhibited a decline across all age groups. In the high-middle SDI regions, the age-specific mortality rate and DALYs rate remained stable for the 80–84 and over 90 age groups, while an increase was observed in the 85–89 age group. For the remaining age groups, both the age-specific mortality rate and DALYs rate exhibited a downward trend. In the middle SDI regions, the age-specific mortality rate increased among individuals over 75 and decreased among those aged 25–69. Similarly, the age-specific DALYs rate exhibited an increase among individuals over 70 and a decline among those aged 25 to 64. In the Low-middle SDI regions, the age-specific mortality rate and DALYs rate exhibited a downward trend among individuals aged 25–34, while they showed an upward trend among those over 40. In the Low SDI regions, the age-specific mortality rate and DALYs rate declined among individuals aged 25–79, but increased among those over 80 (Figures 4A,B).

**Figure 5:**
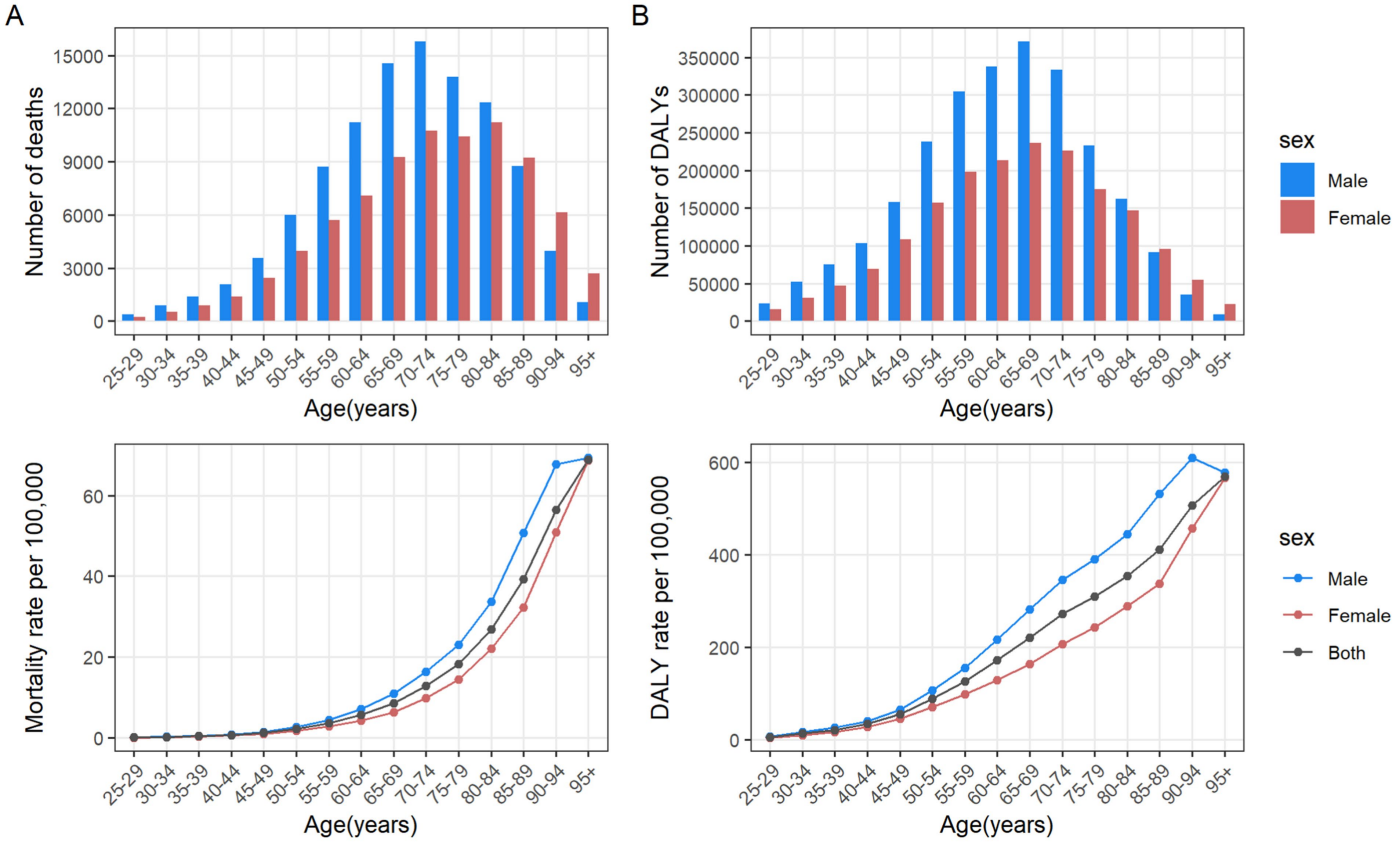
Number and rate of colorectal cancer deaths **(A)** and DALYs **(B)** attributable to diet low in whole grains by age group and sex in the global in 2021. The bars represent the number of colorectal cancer deaths **(A)** and DALYs (B) attributable to diet low in whole grains among different age groups and genders. The line represents the rates of mortality **(A)** and DALYs **(B)** of colorectal cancer due to diet low in whole grains among different age groups and genders. DALYs, Disability-adjusted life years.

**Figure 6:**
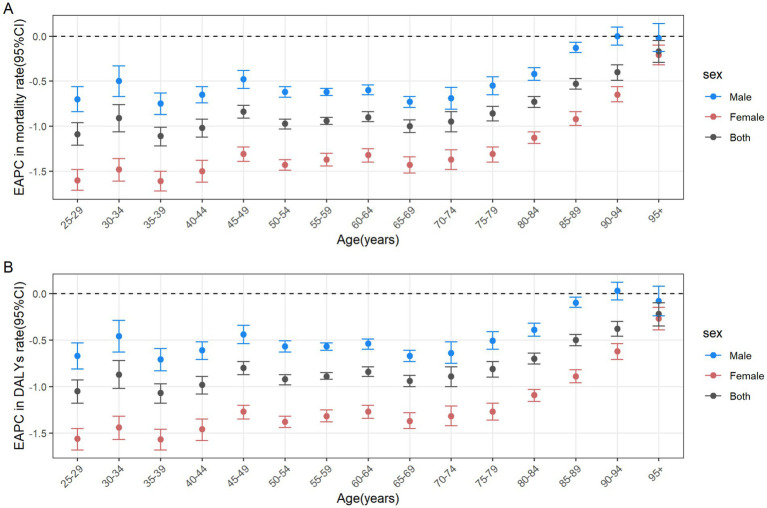
The age distribution of the trends in colorectal cancer mortality rate **(A)** and DALYs rate **(B)** attributable to diet low in whole grains from 1990 to 2021 among different genders. DALYs, Disability-adjusted life years; EAPC, Estimated annual percentage changes; CI, confidence interval.

From 1990 to 2021, the number of male deaths increased from 51,622 (95% UI: 21,646 to 767,288) to 104,344 (95% UI: 42,110 to 159,294), representing a growth rate of 102.13%. Similarly, the number of female deaths increased from 50,191 (95% UI: 20,942 to 74,790) to 81,912 (95% UI: 34,184 to 123,364), representing a 63.20% rise (Table 1). From 1990 to 2021, the average annual decrease in ASMR of CRC attributable to diet low in whole grains was −0.5(95% CI: −0.53 to −0.48) for males, and the average annual decrease was −1.21 (95% CI: −1.26 to −1.16) for females (Table 1). In 2021, globally, the number of CRC deaths and DALYs attributable to diet low in whole grains was consistently higher among males than females in the 25–84 age group, with the reverse pattern observed for those aged 85 and above. The peak number of deaths was reached among males aged 70–74 and females aged 80–84 (Figure 5A). The number of DALYs follows a normal distribution, peaking in the 65–69 age group for both males and females (Figure 5B). The age-specific mortality rate and DALYs rate per 100,000 cases of CRC attributable to diet low in whole grains exhibit an upward trend with advancing age, and consistently demonstrate higher values in males compared to females in 2021 (Figures 5A,B). Among males, from 1990 to 2021, the age-specific mortality rate and DALYs rate declined in the 25–89 age group, while remaining stable in the 90+ age group. The most significant decline was observed in the 35–39 age group (Figures 6A,B). In the case of females, a decline was observed in the age-specific mortality rate and DALYs rate across all age groups, with the most notable decrease also occurring in the 35–39 age group (Figures 6A,B).

### 3.3 The association between the SDI and the CRC burden attributable to diet low in whole grains

**Figure 7:**
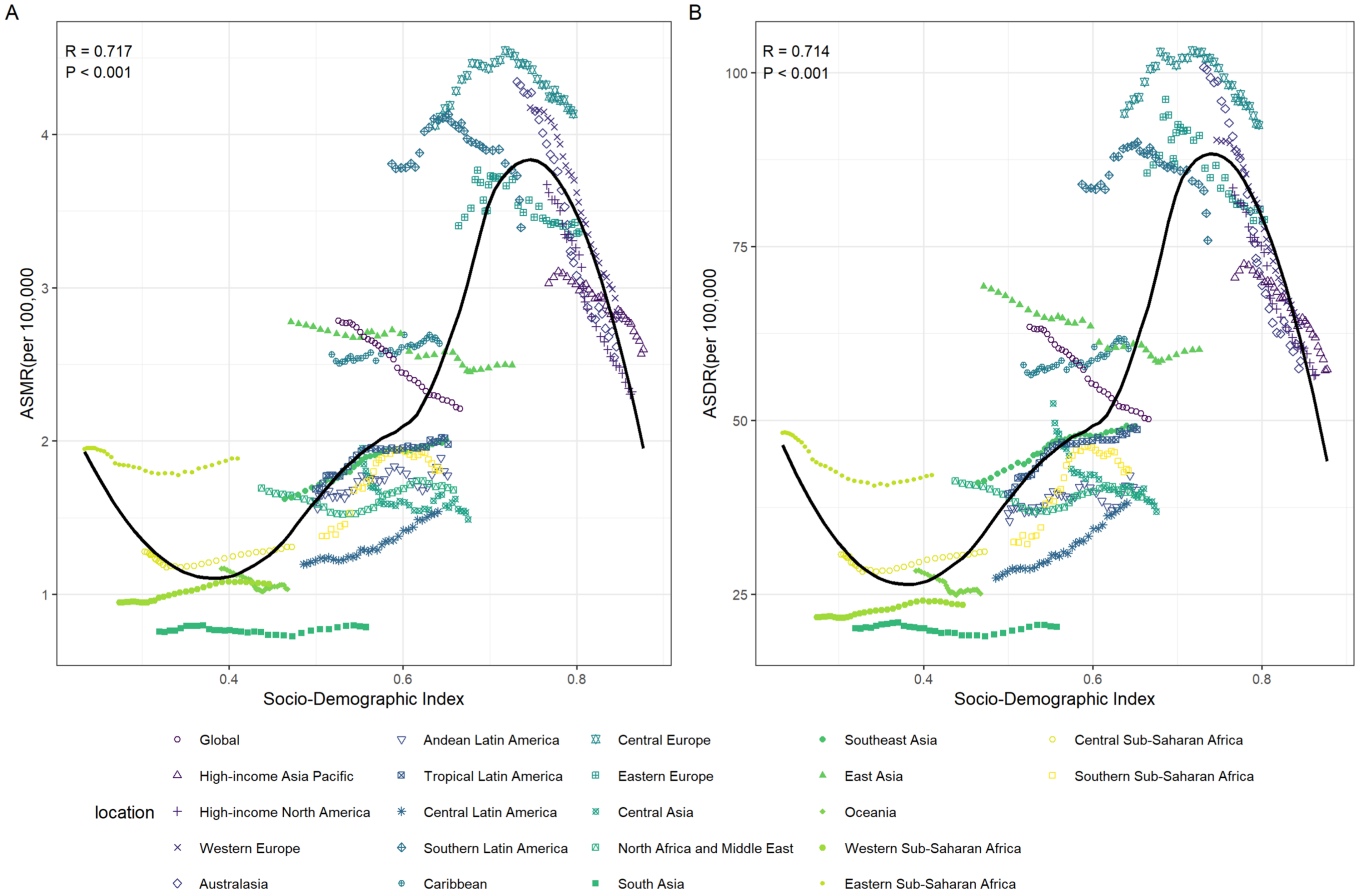
The relationships between the SDI and the colorectal cancer burdens attributable to diet low in whole grains among the 21 GBD regions between 1990 and 2021. The association between colorectal cancer attributable to diet low in whole grains ASMR and SDI among 21 GBD regions **(A)**. The association between colorectal cancer attributable to diet low in whole grains ASDR and SDI among 21 GBD regions **(B)**. ASMR, Age-standardized mortality rate; ASDR, Age-standardized DALY rate; SDI, Socio-demographic index; GBD: global burden of disease.

In general, there was a nonlinear “S”-shaped correlation between SDI and the ASMR and ASDR of CRC attributable to diet low in whole grains. When SDI was between 0.39 and 0.74, both ASMR and ASDR showed an increasing trend, peaking at an SDI of approximately 0.74. This suggested that the greatest burden of death and disability due to CRC occurs at middle to high levels of SDI. Conversely, when SDI was greater than 0.74, both ASMR and ASDR decreased significantly. Among the different regions, Central Europe had the highest ASMR and ASDR of CRC attributable to diet low in whole grains, while South Asia had the lowest (Figures 7A,B).

**Figure 8:**
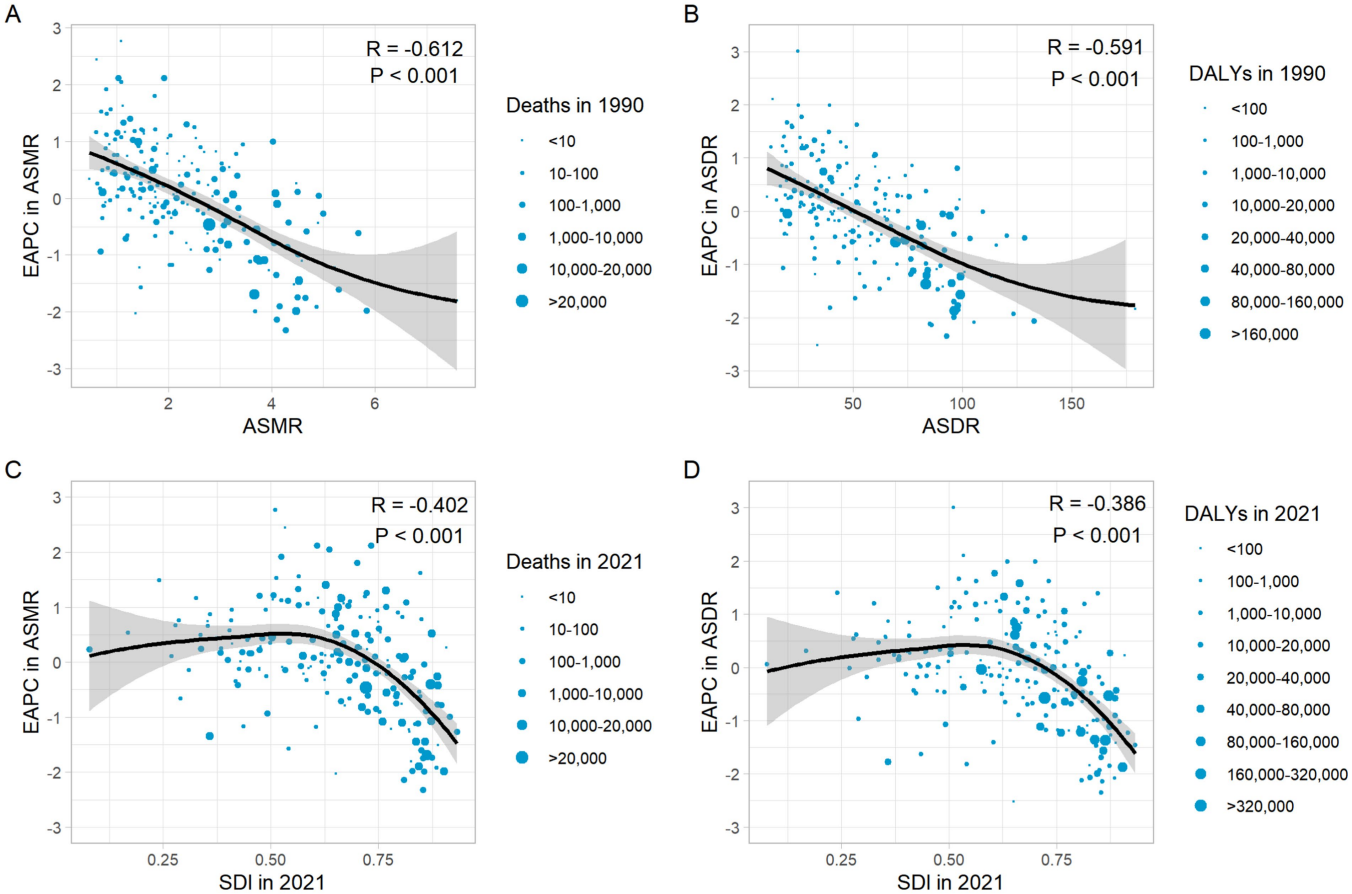
The correlation between EAPC in ASMR and ASMR in 1990 **(A)**. The correlation between EAPC in ASDR and ASDR in 1990 **(B)**. The correlation between EAPC in ASMR and SDI in 2021 **(C)**. The correlation between EAPC in ASDR and SDI in 2021 **(D)**. ASMR, Age-standardized mortality rate; ASDR, Age-standardized DALY rate; DALYs, Disability-adjusted life years; SDI, Socio-demographic index.

The EAPC in ASMR and ASMR in 1990 showed a significant negative correlation (*R* = −0.612, *p* < 0.001) (Figure 8A), as well as the EAPC in ASDR and ASDR in 1990 (*R* = −0.591, *p* < 0.001) (Figure 8B). In 2021, the EAPC in ASMR of CRC attributable to diet low in whole grains was negatively associated with SDI (*R* = −0.402, *p* < 0.001), reaching the highest EAPC at approximately SDI of 0.51 and the lowest at 0.85 (Figure 8C). Similarly, the correlation between EAPC in ASDR and SDI in 2021 exhibited a similar pattern (Figure 8D).

## 4 Discussion

Dietary risk factors have a significant impact on population health and are among the primary risk factors contributing to global diseases and deaths. The GBD 2017 Diet Collaborators revealed that inadequate whole grains intake is one of the leading dietary risk factors for the increase in global DALYs (23). In this study, we conducted an analysis of global, regional, and national spatio-temporal trends in mortality and DALY of CRC attributable to diet low in whole grains.

It was found that from 1990 to 2021, the ASMR and ASDR of CRC attributable to diet low in whole grains declined globally, with a faster decline observed in females compared to males. However, the absolute number of deaths and DALYs increased annually by 82.94 and 70.30% respectively, this increase posing a persistent health challenge. Population growth and changes in age structure may account for this increase. In addition, among people aged 25–84 years, the global burden of CRC deaths and DALYs attributable to diet low in whole grains was significantly higher in males than in females, in contrast to the situation among people aged 85 years and older.

This burden was closely related to socioeconomic development, and notable differences exist in the spatial distribution of CRC burden across different countries and regions. Over the past three decades, the burden of CRC attributable to diet low in whole grains has been relatively high in high, high-middle, and middle SDI regions, especially in East Asia, Western Europe, and High-income North America. However, their ASMR and ASDR have shown a declining trend, with the most significant decline observed in high SDI regions. This may be attributed to a multitude of factors. For example, cultural practices and dietary habits unique to these regions play a crucial role. In East Asia, the high consumption of processed meats and refined grains, coupled with a lower intake of whole grains, likely contributes to the elevated CRC burden (24–26). Additionally, these variations could be influenced by socio-economic and demographic factors, including access to healthcare, smoking rates, alcohol consumption, and levels of physical activity (27). Despite initially higher CRC burdens, high SDI regions may benefit from advancements in healthcare, which have led to observed declines in ASMR and ASDR. In contrast, some less developed regions, including Southern Sub-Saharan Africa, Central Latin America and Western Sub-Saharan Africa, had maintained a relatively low number of deaths and DALYs of CRC attributable to diet low in whole grains. Yet, the ASMR and ASDR in these regions were gradually increasing. In 2021, Uruguay had the highest ASMR, while Bangladesh had the lowest. China ranked 132nd among the 204 countries and regions. In addition, a significant negative correlation was observed between the EAPC in ASMR and the ASMR in 1990. This highlights that even countries with initially lower mortality rates may experience a rapid increase in mortality over time. These findings have important clinical implications, raising public awareness of the importance of increasing whole grains consumption and providing a basis for policymakers to formulate targeted dietary strategies to more effectively address the issue of CRC.

Whole grain foods encompass a wide array of potential cancer-fighting compounds, including antioxidants, trace minerals, phytates, phenolic acids, phytoestrogens and fiber (28). Many studies of whole grains consumption found a negative association between CRC risk and whole grains intake (29–31). However, the mechanism underlying the reduced risk of CRC associated with increased whole grains consumption remains unclear. In intestinal diseases, the primary components of whole grains that exert effects are dietary fiber and polyphenols (32). Dietary fiber has been demonstrated to promote intestinal motility and increases the frequency of bowel movements, thereby diluting and reducing toxins and harmful substances in the intestine (6). A meta-analysis of 25 prospective studies found that for every 10 g/day increase in dietary fiber intake, there is a 10% reduction in the incidence of colorectal cancer (17). Whole grains polyphenols possess antioxidant and anti-inflammatory properties, capable of scavenging free radicals in the body and inhibiting the proliferation of tumor cells (33, 34). Moreover, there may be a dose–response relationship between the inhibitory effects of these substances on colon cancer cells (35). The more whole grains consumed, the more dietary fiber, polyphenols, and other plant-based active ingredients from whole grains are ingested. When these components accumulate to a certain level in the human body, they can more effectively exert their anticancer effects.

The Dietary Guidelines for Americans (2020–2025) recommend a daily intake of six ounce-equivalents of grains, with a minimum of half of this quantity derived from whole grains sources (36). Nevertheless, the entire American population, regardless of age, continues to fall short of the recommended intake of at least one serving of whole grains per day (37). This is primarily due to the gradual premiumization of consumption trends in developed countries, where there is a preference for high-protein, high-nutrition animal-based products. While the burden is concentrated mainly in high-SDI countries, these countries have seen an increase in whole grains consumption in recent decades, primarily attributed to the formulation of relevant dietary guidelines (38, 39).

As a populous country, China also has the highest number of CRC deaths and DALYs attributable to diet low in whole grains (40). In China, although grains have historically been regarded as a traditional dietary staple, there has been a notable decline in the consumption of whole grains among the Chinese population since 1982. Conversely, there has been a steady increase in the consumption of refined grains during the same period (41). Statistical data indicates that the mean daily consumption of whole grains among Chinese residents in 2018 was only 20.1 grams, a figure that falls significantly below the recommended range of 50–150 grams as outlined in the Dietary Guidelines for Chinese Residents (42). The primary factors contributing to this situation are the rapid economic development and changes in dietary patterns, especially the increased consumption of processed foods and fast food, which has resulted in a notable decline in the consumption of traditional whole grains diets. This phenomenon is particularly prominent in developing countries. In some less developed countries in Africa, the Middle East, Latin America and Southeast Asia, the intake of whole grains is deemed to be adequate. However, it is concerning that the ASMR and ASDR in these countries are showing an upward trend year by year, which is closely related to the lagging medical conditions in these regions. It is therefore recommended that policymakers in regions such as Africa, the Middle East, Latin America and Southeast Asia should prioritize the promotion of innovation and advancement in medical technology. At the present time, the consumption of whole grains is more influenced by traditional food consumption habits in specific countries or regions than by adherence to dietary recommendations for whole grains or consumers’ in-depth understanding of the health benefits of whole grains (43). In light of the aforementioned considerations, it is imperative that strategies designed to promote whole grains diets take into account the local cultural context and dietary habits in order to ensure effective implementation.

In 2021, the global burden of CRC attributable to diet low in whole grains was higher among individuals aged 50 to 94, and the age-specific mortality rate and DALYs rate increased with age. This may be due to the decline in general physical condition with age, leading to difficulties in treatment and health management. Additionally, older adults generally have lower whole grains intake compared to younger people due to smaller appetite and weaker digestive function. Therefore, prevention and treatment efforts should be strengthened for the elderly population. It is important to note that due to factors such as being close to the average life expectancy, tolerance for surgical interventions, and the threat of other diseases, elderly individuals should not blindly pursue survival time but should instead prioritize their quality of life.

The findings of our research indicate that the age-specific mortality rate and DALYs rate of CRC attributable to diet low in whole grains are higher in males than in females across all age groups. The precise aetiology of this gender disparity remains unclear; however, genetic and environmental factors are considered to be crucial (44). Compared to females, males typically have diets that encompass a higher prevalence of cancer risk factors, including alcohol consumption, tobacco use, and a higher intake of red meat. Conversely, females tend to engage in less smoking and drinking and consume a larger quantity of fruits and vegetables (45, 46). Studies have also shown that the difference in the incidence and mortality rates of CRC between the sexes is attributed to the role of female estrogens, and relevant epidemiological research results indicate that estrogen replacement therapy can prevent CRC (47, 48). Therefore, we recommend strengthening CRC prevention education and advocating for healthy diets among males, and encouraging females to continue maintaining healthy eating habits, in order to reduce CRC incidence and mortality rates.

To effectively address the escalating burden of colorectal cancer (CRC) attributable to insufficient whole grains intake, a multifaceted and approach tailored to regions with varying levels of development must be implemented. In High SDI and High-middle SDI regions, efforts must involve raising public awareness of the health benefits of whole grains to foster increased daily consumption. Additionally, there is a need to develop scientifically sound and practical dietary guidelines and policies to support these initiatives. Specifically, since processed food consumption is typically higher in these regions, policies should prioritize reducing the intake of such foods and promoting healthier alternatives, such as whole grains. For less developed countries, it is imperative to strengthen healthcare systems, particularly by enhancing early screening and diagnostic capabilities for CRC, to ensure that patients receive timely and effective treatment. Additionally, it is crucial to enhance society’s awareness and acceptance of healthy lifestyles through education and media campaigns, with special attention and guidance given to elderly populations and males.

There are several limitations in this study. First, the data for this study were derived from the GBD 2021. Data collected from low-income countries may vary considerably in accuracy and completeness, potentially impacting the reliability of our findings in these regions. Although the GBD 2021 encompasses a wide range of countries over an extensive time period, it relies on complex estimation and predictive models, thereby introducing a degree of uncertainty. Second, regarding the statistical methodology, the EAPC calculation assumes a linear trend in the data, which may not accurately capture complex or non-linear changes over time, especially when examining long-term health outcome trends. Third, the study, constrained by the limitations of the GBD database, does not account for other dietary factors such as red meat and alcohol consumption, which may interact with whole grains intake and influence colorectal cancer risk. This omission could lead to underestimations or overestimations of the burden attributable to diet low in whole grains.

## 5 Conclusion

Despite a decline in the ASMR and ASDR of CRC attributable to diet low in whole grains from 1990 to 2021 on a global scale, the absolute number of cases continues to increase. It is noteworthy that High-middle SDI regions have the highest ASDR and ASMR of CRC attributable to diet low in whole grains in 2021. Furthermore, Low-middle SDI regions are the only ones where both the ASMR and ASDR have shown an upward trend over the past three decades. The study also revealed significant age- and gender-based disparities, indicating that older adults and males may be at an elevated risk of CRC mortality due to insufficient whole grains consumption.

In summary, this finding not only prompts a profound reflection on the current status of CRC prevention and control globally but also serves as a clear guidance for the formulation and implementation of future health promotion strategies. To effectively address this public health challenge, comprehensive measures and precisely targeted interventions are required. Specifically, longitudinal studies should be conducted to assess the impact of increased whole grains consumption on CRC incidence and mortality. In terms of public health interventions, several strategies can be considered to boost whole grains intake. These include public education campaigns to raise awareness about the benefits of whole grains, subsidies for whole grains products to make them more affordable, and changes to food labeling regulations to highlight whole grains content. By adopting these and other innovative approaches, we can work together to mitigate the burden of CRC related to inadequate whole grains consumption.

## Data Availability

The original contributions presented in the study are included in the article/Supplementary material, further inquiries can be directed to the corresponding author.
