# Supplementary material for: The burden of colorectal cancer attributable to diet low in whole grains from 1990 to 2021: a global, regional and national analysis
Source: Front Nutr. 2025 Apr 9;12:1527522. doi: 10.3389/fnut.2025.1527522 (PMC12014444; doi:10.3389/fnut.2025.1527522)
Supplement: Supplementary file 1 [file Table_1.docx]

Table S1. Deaths and DALYs of CRC attributable to diet low in whole grains in 1990 and 2019 for both sexes and 204 countries, with estimated annual percentage change from 1990 to 2019.

| **Location** | **1990** | | | | **2021** | | | | **EAPC(1990-2021)** | |
| --- | --- | --- | --- | --- | --- | --- | --- | --- | --- | --- |
|  | **Deaths Cases** | **ASMR**  **(/100k)** | **DALYs** | **ASDR**  **(/100k)** | **Deaths Cases** | **ASMR**  **(/100k)** | **DALYs** | **ASDR**  **(/100k)** | **ASMR** | **ASDR** |
|  | **No.**  **(95% UI)** | **No.**  **(95% UI)** | **No.**  **(95% UI)** | **No.**  **(95% UI)** | **No.**  **(95% UI)** | **No.**  **(95% UI)** | **No.**  **(95% UI)** | **No.**  **(95% UI)** | **No.**  **(95% CI)** | **No.**  **(95% CI)** |
| Afghanistan | 150  (43 to 288) | 2.24 (0.68 to 4.21) | 4,340 (1,154 to 8,447) | 60.21 (16.57 to 115.48) | 228 (77 to 430) | 2.32 (0.82 to 4.31) | 7,412 (2,480 to 14,321) | 60.82 (20.57 to 116.92) | 0.25 (0.18 to 0.32) | 0.14 (0.08 to 0.2) |
| Albania | 27 (11 to 42) | 1.46 (0.59 to 2.22) | 659 (271 to 1,008) | 31.65 (12.93 to 48.42) | 62 (26 to 98) | 1.43 (0.59 to 2.27) | 1,271 (527 to 1,997) | 29.61 (12.29 to 46.61) | 0.26 (0.05 to 0.48) | 0.13 (-0.07 to 0.33) |
| Algeria | 96 (39 to 149) | 1.01 (0.41 to 1.54) | 2,559 (1,005 to 3,919) | 21.36 (8.54 to 32.5) | 271 (106 to 423) | 0.91 (0.37 to 1.42) | 6,710 (2,539 to 10,536) | 18.81 (7.29 to 29.48) | 0.03 (-0.14 to 0.20) | -0.24 (-0.35 to -0.13) |
| American Samoa | 1 (0 to 1) | 2.77 (1.14 to 4.36) | 16 (6 to 25) | 64.85 (26.18 to 101.09) | 1 (0 to 2) | 2.85 (1.15 to 4.37) | 33 (13 to 51) | 66.99 (26.74 to 104.41) | 0.23 (0.12 to 0.35) | 0.23 (0.12 to 0.34) |
| Andorra | 3 (1 to 4) | 4.58 (1.82 to 7.43) | 59 (23 to 96) | 101.07 (39.62 to 165.73) | 5 (2 to 8) | 2.96 (1.22 to 4.87) | 98 (39 to 162) | 64.01 (25.75 to 105.42) | -1.10 (-1.35 to -0.85) | -1.15 (-1.38 to -0.91) |
| Angola | 52 (21 to 85) | 1.46 (0.61 to 2.38) | 1,553 (645 to 2,573) | 35.91 (15.02 to 59.26) | 161 (61 to 262) | 1.53 (0.59 to 2.52) | 4,789 (1,791 to 7,803) | 36.36 (13.79 to 59.4) | 0.16 (0.06 to 0.27) | 0.05 (-0.06 to 0.17) |
| **Location** | **1990** | | | | **2021** | | | | **EAPC(1990-2021)** | |
|  | **Deaths Cases** | **ASMR**  **(/100k)** | **DALYs** | **ASDR**  **(/100k)** | **Deaths Cases** | **ASMR**  **(/100k)** | **DALYs** | **ASDR**  **(/100k)** | **ASMR** | **ASDR** |
|  | **No.**  **(95% UI)** | **No.**  **(95% UI)** | **No.**  **(95% UI)** | **No.**  **(95% UI)** | **No.**  **(95% UI)** | **No.**  **(95% UI)** | **No.**  **(95% UI)** | **No.**  **(95% UI)** | **No.**  **(95% CI)** | **No.**  **(95% CI)** |
| Antigua and Barbuda | 1 (1 to 2) | 2.31 (0.95 to 3.45) | 27 (11 to 41) | 51.55 (21.21 to 76.76) | 3 (1 to 4) | 2.83 (1.18 to 4.17) | 65 (27 to 95) | 60.27 (25.00 to 89.13) | 0.74 (0.55 to 0.92) | 0.61 (0.45 to 0.76) |
| Argentina | 1,282 (533 to 1,930) | 4.11 (1.71 to 6.16) | 29,382 (12,131 to 44,397) | 91.08 (37.69 to 137.56) | 2,074 (854 to 3,195) | 3.64 (1.50 to 5.61) | 45,425 (18,711 to 70,168) | 82.46 (34.02 to 127.64) | -0.11 (-0.32 to 0.10) | -0.05 (-0.24 to 0.15) |
| Armenia | 69 (29 to 102) | 2.57 (1.1 to 3.82) | 1,954 (834 to 2,895) | 67.47 (28.78 to 99.93) | 108 (46 to 158) | 2.49 (1.06 to 3.64) | 2,446 (1,037 to 3,597) | 56.78 (24.03 to 83.47) | 0.28 (0.07 to 0.48) | -0.29 (-0.48 to -0.1) |
| Australia | 804 (333 to 1,222) | 4.15 (1.73 to 6.33) | 18,587 (7,722 to 28,159) | 96.3 (40.05 to 146.00) | 1,189 (483 to 1,810) | 2.49 (1.03 to 3.79) | 24,068 (10,149 to 36,689) | 56.26 (24.05 to 85.41) | -1.89 (-1.99 to -1.80) | -2.00 (-2.10 to -1.89) |
| Austria | 522 (223 to 784) | 4.28 (1.83 to 6.41) | 10,689 (4,541 to 16,066) | 93.03 (39.49 to 139.72) | 415 (168 to 634) | 2.09 (0.84 to 3.19) | 7,840 (3,139 to 11,912) | 44.69 (17.80 to 67.90) | -2.32 (-2.37 to -2.27) | -2.35 (-2.40 to -2.30) |
| Azerbaijan | 83 (37 to 127) | 1.67 (0.74 to 2.56) | 2,501 (1,106 to 3,836) | 46.06 (20.38 to 70.63) | 135 (55 to 212) | 1.36 (0.54 to 2.13) | 3,860 (1,572 to 6,106) | 34.61 (14.10 to 54.27) | -0.24 (-0.51 to 0.04) | -0.68 (-0.93 to -0.42) |
| **Location** | **1990** | | | | **2021** | | | | **EAPC(1990-2021)** | |
|  | **Deaths Cases** | **ASMR**  **(/100k)** | **DALYs** | **ASDR**  **(/100k)** | **Deaths Cases** | **ASMR**  **(/100k)** | **DALYs** | **ASDR**  **(/100k)** | **ASMR** | **ASDR** |
|  | **No.**  **(95% UI)** | **No.**  **(95% UI)** | **No.**  **(95% UI)** | **No.**  **(95% UI)** | **No.**  **(95% UI)** | **No.**  **(95% UI)** | **No.**  **(95% UI)** | **No.**  **(95% UI)** | **No.**  **(95% CI)** | **No.**  **(95% CI)** |
| Bahamas | 4 (2 to 7) | 2.92 (1.24 to 4.38) | 123 (52 to 185) | 74.02 (31.20 to 111.29) | 13 (5 to 21) | 3.47 (1.42 to 5.42) | 348 (140 to 546) | 83.02 (33.51 to 130.23) | 0.82 (0.69 to 0.95) | 0.67 (0.56 to 0.77) |
| Bahrain | 3 (1 to 5) | 2.44 (0.99 to 3.74) | 103 (43 to 159) | 53.08 (21.59 to 81.74) | 14 (6 to 22) | 2.07 (0.84 to 3.21) | 413 (162 to 663) | 42.6 (17.02 to 66.62) | -0.9 (-1.21 to -0.59) | -1.17 (-1.43 to -0.92) |
| Bangladesh | 321 (134 to 521) | 0.69 (0.29 to 1.13) | 9,448 (3,896 to 15,179) | 18.28 (7.59 to 29.44) | 702 (266 to 1,159) | 0.53 (0.20 to 0.86) | 18,811 (7,160 to 31,947) | 13.09 (5.01 to 22.04) | -0.93 (-1.07 to -0.80) | -1.07 (-1.17 to -0.97) |
| Barbados | 10 (4 to 16) | 3.44 (1.43 to 5.23) | 217 (90 to 332) | 77.15 (32.05 to 117.51) | 22 (8 to 35) | 4.23 (1.54 to 6.68) | 461 (167 to 727) | 91.03 (33.04 to 143.52) | 0.95 (0.69 to 1.20) | 0.86 (0.63 to 1.08) |
| Belarus | 397 (162 to 602) | 3.07 (1.25 to 4.66) | 10,017 (4,101 to 15,202) | 77.3 (31.65 to 117.24) | 533 (196 to 846) | 3.28 (1.21 to 5.20) | 12,483 (4,589 to 19,753) | 78.58 (28.87 to 124.24) | -0.48 (-0.78 to -0.17) | -0.69 (-0.99 to -0.39) |
| Belgium | 733 (314 to 1,083) | 4.65 (2.00 to 6.89) | 14,531 (6,201 to 21,453) | 96.16 (41.08 to 142.13) | 679 (280 to 1,031) | 2.61 (1.07 to 3.95) | 12,560 (5,058 to 18,930) | 55.65 (22.31 to 83.83) | -1.75 (-1.84 to -1.65) | -1.70 (-1.81 to -1.59) |
| **Location** | **1990** | | | | **2021** | | | | **EAPC(1990-2021)** | |
|  | **Deaths Cases** | **ASMR**  **(/100k)** | **DALYs** | **ASDR**  **(/100k)** | **Deaths Cases** | **ASMR**  **(/100k)** | **DALYs** | **ASDR**  **(/100k)** | **ASMR** | **ASDR** |
|  | **No.**  **(95% UI)** | **No.**  **(95% UI)** | **No.**  **(95% UI)** | **No.**  **(95% UI)** | **No.**  **(95% UI)** | **No.**  **(95% UI)** | **No.**  **(95% UI)** | **No.**  **(95% UI)** | **No.**  **(95% CI)** | **No.**  **(95% CI)** |
| Belize | 1 (0 to 2) | 1.17 (0.48 to 1.76) | 26 (10 to 39) | 26.98 (11.03 to 40.90) | 5 (2 to 7) | 1.66 (0.64 to 2.51) | 125 (48 to 187) | 39.37 (15.28 to 59.1) | 1.16 (0.69 to 1.64) | 1.24 (0.79 to 1.7) |
| Benin | 17 (7 to 25) | 0.90 (0.40 to 1.36) | 419 (184 to 641) | 20.53 (9.07 to 31.40) | 44 (17 to 71) | 0.98 (0.39 to 1.55) | 1,143 (446 to 1,870) | 21.23 (8.32 to 34.29) | 0.44 (0.35 to 0.53) | 0.24 (0.14 to 0.33) |
| Bermuda | 3 (1 to 5) | 5.22 (2.16 to 7.86) | 71 (30 to 108) | 113.38 (47.18 to 172.78) | 5 (2 to 8) | 3.42 (1.26 to 5.36) | 98 (36 to 154) | 73.55 (27.33 to 116.36) | -1.22 (-1.39 to -1.05) | -1.23 (-1.40 to -1.06) |
| Bhutan | 2 (1 to 3) | 0.77 (0.28 to 1.26) | 56 (20 to 93) | 20.08 (7.34 to 33.25) | 4 (2 to 7) | 0.74 (0.28 to 1.20) | 113 (43 to 185) | 17.88 (6.71 to 29.01) | -0.14 (-0.24 to -0.03) | -0.45 (-0.55 to -0.34) |
| Bolivia (Plurinational State of) | 73 (28 to 117) | 2.49 (0.95 to 3.96) | 1,917 (713 to 3,076) | 57.44 (21.58 to 91.88) | 212 (78 to 357) | 2.53 (0.94 to 4.24) | 5,135 (1,878 to 8,679) | 55.37 (20.21 to 93.43) | 0.08 (0.05 to 0.11) | -0.15 (-0.19 to -0.10) |
| Bosnia and Herzegovina | 96 (41 to 143) | 2.5 (1.06 to 3.71) | 2,536 (1,085 to 3,764) | 59.65 (25.44 to 88.15) | 213 (82 to 333) | 3.33 (1.28 to 5.20) | 4,585 (1,775 to 7,216) | 74.42 (28.71 to 117.21) | 1.26 (1.08 to 1.44) | 1.04 (0.83 to 1.25) |
| Botswana | 9 (4 to 14) | 1.74 (0.74 to 2.78) | 237 (103 to 382) | 40.52 (17.42 to 65.11) | 23 (9 to 36) | 1.76 (0.70 to 2.84) | 617 (238 to 1,011) | 39.56 (15.32 to 63.41) | 0.12 (-0.09 to 0.33) | -0.05 (-0.28 to 0.17) |
| **Location** | **1990** | | | | **2021** | | | | **EAPC(1990-2021)** | |
|  | **Deaths Cases** | **ASMR**  **(/100k)** | **DALYs** | **ASDR**  **(/100k)** | **Deaths Cases** | **ASMR**  **(/100k)** | **DALYs** | **ASDR**  **(/100k)** | **ASMR** | **ASDR** |
|  | **No.**  **(95% UI)** | **No.**  **(95% UI)** | **No.**  **(95% UI)** | **No.**  **(95% UI)** | **No.**  **(95% UI)** | **No.**  **(95% UI)** | **No.**  **(95% UI)** | **No.**  **(95% UI)** | **No.**  **(95% CI)** | **No.**  **(95% CI)** |
| Brazil | 1,404 (581 to 2,102) | 1.70 (0.71 to 2.57) | 37,450 (15,454 to 56,040) | 39.78 (16.43 to 59.66) | 4,926 (2,094 to 7,433) | 1.98 (0.84 to 2.99) | 124,464 (53,095 to 187,650) | 48.87 (20.84 to 73.66) | 0.50 (0.42 to 0.58) | 0.62 (0.53 to 0.71) |
| Brunei Darussalam | 5 (2 to 7) | 4.9 (1.97 to 7.74) | 135 (55 to 212) | 115.56 (46.35 to 180.52) | 11 (4 to 17) | 3.55 (1.45 to 5.57) | 316 (127 to 499) | 82.09 (33.22 to 128.64) | -0.44 (-0.72 to -0.15) | -0.64 (-0.92 to -0.36) |
| Bulgaria | 473 (196 to 715) | 4.03 (1.67 to 6.08) | 11,993 (4,968 to 18,178) | 97.78 (40.48 to 147.5) | 700 (272 to 1,086) | 4.80 (1.87 to 7.44) | 15,204 (5,844 to 23,605) | 112.6 (43.12 to 175.89) | 1.00 (0.81 to 1.19) | 0.8 (0.65 to 0.96) |
| Burkina Faso | 36 (15 to 58) | 0.94 (0.39 to 1.5) | 942 (376 to 1,529) | 21.37 (8.69 to 34.51) | 91 (35 to 148) | 1.12 (0.44 to 1.79) | 2,320 (883 to 3,830) | 24.27 (9.36 to 39.65) | 0.76 (0.69 to 0.84) | 0.61 (0.52 to 0.70) |
| Burundi | 38 (15 to 58) | 1.74 (0.68 to 2.65) | 1,073 (421 to 1,658) | 43.47 (17.11 to 66.8) | 67 (26 to 113) | 1.55 (0.61 to 2.56) | 1,910 (754 to 3,269) | 35.77 (14.14 to 60.35) | -0.66 (-0.80 to -0.53) | -0.96 (-1.13 to -0.8) |
| Cabo Verde | 1 (1 to 2) | 0.62 (0.26 to 0.95) | 30 (13 to 46) | 13.42 (5.76 to 20.59) | 6 (2 to 10) | 1.56 (0.60 to 2.48) | 135 (50 to 217) | 30.59 (11.29 to 49.28) | 2.44 (2.03 to 2.86) | 2.11 (1.71 to 2.51) |
| Cambodia | 83 (33 to 136) | 1.91 (0.77 to 3.12) | 2,486 (968 to 4,118) | 49.39 (19.51 to 81.12) | 240 (91 to 390) | 2.06 (0.77 to 3.39) | 6,728 (2,615 to 11,117) | 50.42 (19.36 to 82.87) | 0.22 (0.15 to 0.30) | 0.01 (-0.05 to 0.07) |
| **Location** | **1990** | | | | **2021** | | | | **EAPC(1990-2021)** | |
|  | **Deaths Cases** | **ASMR**  **(/100k)** | **DALYs** | **ASDR**  **(/100k)** | **Deaths Cases** | **ASMR**  **(/100k)** | **DALYs** | **ASDR**  **(/100k)** | **ASMR** | **ASDR** |
|  | **No.**  **(95% UI)** | **No.**  **(95% UI)** | **No.**  **(95% UI)** | **No.**  **(95% UI)** | **No.**  **(95% UI)** | **No.**  **(95% UI)** | **No.**  **(95% UI)** | **No.**  **(95% UI)** | **No.**  **(95% CI)** | **No.**  **(95% CI)** |
| Cameroon | 46 (18 to 70) | 1.16 (0.47 to 1.76) | 1,229 (494 to 1,873) | 26.55 (10.63 to 40.65) | 140 (57 to 238) | 1.29 (0.54 to 2.11) | 3,842 (1,520 to 6,599) | 28.61 (11.69 to 48.5) | 0.44 (0.37 to 0.51) | 0.33 (0.26 to 0.40) |
| Canada | 1,209 (526 to 1,815) | 3.72 (1.62 to 5.60) | 27,131 (11,742 to 40,800) | 84.29 (36.39 to 126.67) | 1,913 (794 to 2,934) | 2.52 (1.04 to 3.84) | 38,297 (15,861 to 58,580) | 56.45 (23.33 to 86.93) | -1.08 (-1.18 to -0.97) | -1.12 (-1.2 to -1.03) |
| Central African Republic | 18 (8 to 30) | 1.73 (0.73 to 2.86) | 554 (233 to 911) | 43.58 (18.56 to 71.14) | 34 (14 to 59) | 1.65 (0.67 to 2.84) | 1,070 (422 to 1,883) | 41.35 (16.56 to 72.02) | -0.14 (-0.2 to -0.08) | -0.18 (-0.24 to -0.11) |
| Chad | 21 (9 to 34) | 0.81 (0.34 to 1.29) | 533 (219 to 850) | 18.6 (7.67 to 29.83) | 61 (25 to 96) | 1.19 (0.49 to 1.85) | 1,641 (682 to 2,661) | 26.88 (11.18 to 42.66) | 1.49 (1.37 to 1.61) | 1.41 (1.29 to 1.52) |
| Chile | 223 (94 to 335) | 2.36 (1 to 3.55) | 5,227 (2,196 to 7,839) | 51.53 (21.65 to 77.24) | 636 (263 to 958) | 2.45 (1.01 to 3.68) | 13,755 (5,685 to 20,903) | 54.19 (22.44 to 82.27) | 0.42 (0.3 to 0.53) | 0.48 (0.35 to 0.61) |
| China | 21,330 (8,463 to 32,784) | 2.79 (1.11 to 4.29) | 624,948 (248,200 to 959,563) | 69.54 (27.62 to 106.93) | 49,991 (20,100 to 79,929) | 2.47 (0.99 to 3.94) | 1241,928 (503,165 to 1978,508) | 59.7 (24.17 to 94.95) | -0.46 (-0.52 to -0.41) | -0.57 (-0.65 to -0.50) |
| Colombia | 272 (115 to 401) | 1.66 (0.70 to 2.45) | 7,121 (2,982 to 10,460) | 38.13 (16.06 to 56.22) | 1,015 (380 to 1,580) | 1.84 (0.69 to 2.87) | 24,761 (9,383 to 38,408) | 44.96 (17.05 to 69.71) | 0.19 (0.04 to 0.34) | 0.38 (0.23 to 0.54) |
| **Location** | **1990** | | | | **2021** | | | | **EAPC(1990-2021)** | |
|  | **Deaths Cases** | **ASMR**  **(/100k)** | **DALYs** | **ASDR**  **(/100k)** | **Deaths Cases** | **ASMR**  **(/100k)** | **DALYs** | **ASDR**  **(/100k)** | **ASMR** | **ASDR** |
|  | **No.**  **(95% UI)** | **No.**  **(95% UI)** | **No.**  **(95% UI)** | **No.**  **(95% UI)** | **No.**  **(95% UI)** | **No.**  **(95% UI)** | **No.**  **(95% UI)** | **No.**  **(95% UI)** | **No.**  **(95% CI)** | **No.**  **(95% CI)** |
| Comoros | 3 (1 to 5) | 1.63 (0.61 to 2.56) | 85 (31 to 136) | 39.92 (14.71 to 63.8) | 8 (3 to 13) | 1.80 (0.73 to 2.89) | 211 (84 to 345) | 41.21 (16.47 to 66.35) | 0.28 (0.19 to 0.37) | -0.01 (-0.14 to 0.12) |
| Congo | 18 (7 to 30) | 1.90 (0.72 to 3.03) | 534 (201 to 871) | 46.81 (17.50 to 75.74) | 44 (18 to 69) | 1.78 (0.74 to 2.80) | 1,301 (518 to 2,103) | 42.55 (17.24 to 67.48) | -0.32 (-0.43 to -0.21) | -0.45 (-0.58 to -0.33) |
| Cook Islands | 0 (0 to 0) | 1.44 (0.60 to 2.25) | 4 (2 to 7) | 32.75 (13.47 to 51.39) | 0 (0 to 0) | 1.04 (0.41 to 1.59) | 6 (2 to 9) | 23.49 (9.12 to 36.56) | -1.22 (-1.37 to -1.06) | -1.19 (-1.36 to -1.02) |
| Costa Rica | 29 (12 to 44) | 1.73 (0.73 to 2.57) | 714 (300 to 1,065) | 39.39 (16.58 to 58.66) | 152 (61 to 235) | 2.77 (1.10 to 4.27) | 3,710 (1,491 to 5,693) | 67.45 (27.12 to 103.55) | 1.81 (1.61 to 2) | 1.99 (1.78 to 2.20) |
| Croatia | 255 (111 to 387) | 4.50 (1.96 to 6.83) | 5,852 (2,540 to 8,849) | 97.39 (42.24 to 147.07) | 425 (168 to 634) | 4.46 (1.78 to 6.69) | 8,378 (3,361 to 12,675) | 96.27 (38.66 to 145.72) | 0.12 (-0.05 to 0.29) | 0.05 (-0.14 to 0.24) |
| Cuba | 291 (126 to 438) | 2.89 (1.25 to 4.35) | 6,647 (2,873 to 9,979) | 64.84 (28.00 to 97.47) | 632 (243 to 972) | 3.13 (1.21 to 4.81) | 13,299 (5,214 to 20,614) | 69.19 (27.21 to 107.17) | 0.35 (0.23 to 0.46) | 0.31 (0.18 to 0.44) |
| Cyprus | 24 (10 to 37) | 3.77 (1.57 to 5.84) | 521 (223 to 793) | 70.13 (29.89 to 106.99) | 47 (19 to 72) | 2.40 (1.00 to 3.65) | 939 (383 to 1,449) | 46.65 (19.08 to 71.74) | -1.12 (-1.28 to -0.96) | -1.00 (-1.12 to -0.87) |
| **Location** | **1990** | | | | **2021** | | | | **EAPC(1990-2021)** | |
|  | **Deaths Cases** | **ASMR**  **(/100k)** | **DALYs** | **ASDR**  **(/100k)** | **Deaths Cases** | **ASMR**  **(/100k)** | **DALYs** | **ASDR**  **(/100k)** | **ASMR** | **ASDR** |
|  | **No.**  **(95% UI)** | **No.**  **(95% UI)** | **No.**  **(95% UI)** | **No.**  **(95% UI)** | **No.**  **(95% UI)** | **No.**  **(95% UI)** | **No.**  **(95% UI)** | **No.**  **(95% UI)** | **No.**  **(95% CI)** | **No.**  **(95% CI)** |
| Czechia | 814 (338 to 1,242) | 5.84 (2.43 to 8.92) | 18,242 (7,580 to 27,978) | 133.03 (55.19 to 204.18) | 776 (322 to 1,195) | 3.45 (1.43 to 5.32) | 15,858 (6,562 to 24,452) | 76.4 (31.67 to 118.09) | -1.97 (-2.15 to -1.79) | -2.07 (-2.23 to -1.91) |
| Côte d'Ivoire | 26 (11 to 39) | 0.75 (0.31 to 1.13) | 754 (306 to 1,133) | 17.30 (7.14 to 26.02) | 72 (30 to 117) | 0.73 (0.30 to 1.16) | 2,051 (865 to 3,414) | 16.68 (6.92 to 27.25) | -0.13 (-0.22 to -0.03) | -0.18 (-0.28 to -0.07) |
| Democratic People's Republic of Korea | 301 (121 to 517) | 1.96 (0.80 to 3.35) | 8,978 (3,571 to 15,461) | 51.04 (20.58 to 87.77) | 590 (232 to 1,032) | 1.81 (0.71 to 3.16) | 16,379 (6,334 to 28,954) | 48.47 (18.82 to 85.54) | -0.07 (-0.17 to 0.03) | -0.01 (-0.09 to 0.07) |
| Democratic Republic of the Congo | 153 (67 to 249) | 1.11 (0.49 to 1.78) | 4,406 (1,917 to 7,145) | 26.39 (11.78 to 42.79) | 377 (143 to 675) | 1.15 (0.44 to 2.09) | 10,963 (4,131 to 19,504) | 27.22 (10.35 to 48.79) | 0.17 (0.02 to 0.32) | 0.15 (0 to 0.3) |
| Denmark | 339 (140 to 513) | 4.04 (1.67 to 6.10) | 7,019 (2,929 to 10,679) | 89.99 (37.66 to 136.92) | 456 (183 to 693) | 3.53 (1.42 to 5.36) | 8,421 (3,409 to 12,711) | 71.56 (29.03 to 107.56) | -0.78 (-1.09 to -0.48) | -1.03 (-1.32 to -0.73) |
| Djibouti | 2 (1 to 4) | 1.79 (0.70 to 2.92) | 67 (26 to 111) | 42.93 (16.58 to 70.84) | 13 (5 to 23) | 2.37 (0.94 to 3.98) | 383 (149 to 684) | 54 (21.13 to 93.11) | 0.95 (0.88 to 1.03) | 0.76 (0.67 to 0.84) |
| Dominica | 1 (1 to 2) | 2.46 (1.03 to 3.74) | 31 (13 to 47) | 52.87 (21.87 to 80.44) | 2 (1 to 3) | 2.88 (1.16 to 4.36) | 52 (21 to 79) | 62.46 (25.01 to 94.59) | 0.61 (0.55 to 0.68) | 0.67 (0.61 to 0.74) |
| **Location** | **1990** | | | | **2021** | | | | **EAPC(1990-2021)** | |
|  | **Deaths Cases** | **ASMR**  **(/100k)** | **DALYs** | **ASDR**  **(/100k)** | **Deaths Cases** | **ASMR**  **(/100k)** | **DALYs** | **ASDR**  **(/100k)** | **ASMR** | **ASDR** |
|  | **No.**  **(95% UI)** | **No.**  **(95% UI)** | **No.**  **(95% UI)** | **No.**  **(95% UI)** | **No.**  **(95% UI)** | **No.**  **(95% UI)** | **No.**  **(95% UI)** | **No.**  **(95% UI)** | **No.**  **(95% CI)** | **No.**  **(95% CI)** |
| Dominican Republic | 47 (19 to 72) | 1.42 (0.58 to 2.17) | 1,208 (487 to 1,854) | 31.32 (12.7 to 48.14) | 159 (64 to 252) | 1.62 (0.66 to 2.57) | 3,874 (1,522 to 6,062) | 37.97 (14.94 to 59.43) | 0.93 (0.72 to 1.13) | 1.05 (0.92 to 1.19) |
| Ecuador | 66 (27 to 99) | 1.34 (0.56 to 2.03) | 1,576 (657 to 2,381) | 28.91 (12.10 to 43.67) | 272 (109 to 423) | 1.71 (0.69 to 2.65) | 6,417 (2,557 to 9,983) | 38.78 (15.48 to 60.21) | 1.19 (0.87 to 1.5) | 1.21 (0.90 to 1.52) |
| Egypt | 264 (110 to 401) | 1.03 (0.43 to 1.55) | 8,332 (3,453 to 12,600) | 26.51 (11.04 to 40.12) | 902 (352 to 1,404) | 1.59 (0.63 to 2.48) | 26,603 (10,430 to 41,606) | 38.15 (14.88 to 59.57) | 2.12 (1.83 to 2.42) | 1.77 (1.53 to 2.01) |
| El Salvador | 26 (11 to 39) | 0.88 (0.38 to 1.32) | 692 (291 to 1,025) | 22.03 (9.29 to 32.63) | 89 (36 to 143) | 1.41 (0.57 to 2.26) | 2,191 (879 to 3,497) | 35.71 (14.33 to 57.03) | 1.57 (1.39 to 1.74) | 1.62 (1.45 to 1.79) |
| Equatorial Guinea | 3 (1 to 5) | 1.54 (0.59 to 2.45) | 80 (30 to 133) | 38.39 (14.33 to 63.36) | 8 (3 to 14) | 1.78 (0.67 to 3.06) | 241 (89 to 423) | 41.48 (15.41 to 71.58) | 0.63 (0.47 to 0.79) | 0.36 (0.17 to 0.54) |
| Eritrea | 19 (8 to 30) | 1.76 (0.72 to 2.83) | 614 (248 to 997) | 45.32 (18.64 to 72.63) | 51 (21 to 84) | 2.08 (0.87 to 3.34) | 1,566 (634 to 2,629) | 49.44 (20.14 to 81.01) | 0.57 (0.49 to 0.66) | 0.31 (0.23 to 0.38) |
| Estonia | 71 (29 to 107) | 3.46 (1.43 to 5.20) | 1,706 (705 to 2,568) | 83.46 (34.52 to 125.67) | 97 (36 to 150) | 3.25 (1.20 to 5.02) | 1,838 (676 to 2,835) | 69.57 (25.56 to 107.52) | -0.54 (-0.70 to -0.37) | -0.99 (-1.17 to -0.8) |
| **Location** | **1990** | | | | **2021** | | | | **EAPC(1990-2021)** | |
|  | **Deaths Cases** | **ASMR**  **(/100k)** | **DALYs** | **ASDR**  **(/100k)** | **Deaths Cases** | **ASMR**  **(/100k)** | **DALYs** | **ASDR**  **(/100k)** | **ASMR** | **ASDR** |
|  | **No.**  **(95% UI)** | **No.**  **(95% UI)** | **No.**  **(95% UI)** | **No.**  **(95% UI)** | **No.**  **(95% UI)** | **No.**  **(95% UI)** | **No.**  **(95% UI)** | **No.**  **(95% UI)** | **No.**  **(95% CI)** | **No.**  **(95% CI)** |
| Eswatini | 5 (2 to 8) | 1.99 (0.78 to 3.24) | 145 (56 to 238) | 47.11 (18.22 to 77.44) | 13 (5 to 21) | 2.42 (1.00 to 3.99) | 379 (156 to 635) | 59.94 (24.85 to 99.18) | 1.06 (0.63 to 1.49) | 1.21 (0.71 to 1.71) |
| Ethiopia | 716 (274 to 1,154) | 4.09 (1.66 to 6.60) | 20,551 (7,795 to 33,372) | 97.89 (37.85 to 158.13) | 1,121 (467 to 1,723) | 2.94 (1.23 to 4.53) | 28,327 (11,615 to 43,683) | 63.01 (25.99 to 96.50) | -1.35 (-1.53 to -1.16) | -1.77 (-1.97 to -1.57) |
| Fiji | 5 (2 to 8) | 1.65 (0.69 to 2.57) | 163 (68 to 256) | 40.31 (16.75 to 63.21) | 12 (5 to 20) | 1.80 (0.69 to 2.87) | 333 (129 to 530) | 41.7 (16.1 to 66.39) | 0.28 (0.09 to 0.46) | 0.17 (-0.04 to 0.39) |
| Finland | 199 (85 to 305) | 2.74 (1.17 to 4.21) | 4,226 (1,798 to 6,395) | 60.13 (25.64 to 91.03) | 290 (116 to 446) | 2.07 (0.83 to 3.17) | 5,409 (2,179 to 8,258) | 44.57 (18.12 to 68.08) | -0.89 (-1.02 to -0.77) | -0.96 (-1.05 to -0.88) |
| France | 3,928 (1,679 to 5,838) | 4.53 (1.94 to 6.73) | 77,095 (32,904 to 113,639) | 95.09 (40.71 to 140.26) | 4,644 (1,846 to 7,143) | 2.86 (1.14 to 4.38) | 82,598 (33,209 to 125,235) | 61.16 (25.05 to 93.00) | -1.45 (-1.51 to -1.40) | -1.36 (-1.41 to -1.30) |
| Gabon | 12 (4 to 21) | 2.27 (0.74 to 3.91) | 326 (107 to 558) | 55.52 (18.09 to 95.13) | 21 (8 to 35) | 2.24 (0.89 to 3.59) | 596 (236 to 982) | 52.88 (20.95 to 85.95) | -0.08 (-0.13 to -0.02) | -0.19 (-0.25 to -0.13) |
| Gambia | 1 (1 to 2) | 0.47 (0.20 to 0.71) | 41 (16 to 63) | 10.88 (4.45 to 16.81) | 5 (2 to 8) | 0.53 (0.21 to 0.84) | 129 (50 to 205) | 12.31 (4.82 to 19.57) | 0.34 (0.22 to 0.45) | 0.27 (0.11 to 0.42) |
| **Location** | **1990** | | | | **2021** | | | | **EAPC(1990-2021)** | |
|  | **Deaths Cases** | **ASMR**  **(/100k)** | **DALYs** | **ASDR**  **(/100k)** | **Deaths Cases** | **ASMR**  **(/100k)** | **DALYs** | **ASDR**  **(/100k)** | **ASMR** | **ASDR** |
|  | **No.**  **(95% UI)** | **No.**  **(95% UI)** | **No.**  **(95% UI)** | **No.**  **(95% UI)** | **No.**  **(95% UI)** | **No.**  **(95% UI)** | **No.**  **(95% UI)** | **No.**  **(95% UI)** | **No.**  **(95% CI)** | **No.**  **(95% CI)** |
| Georgia | 119 (51 to 180) | 1.91 (0.82 to 2.89) | 3,271 (1,416 to 4,937) | 51.92 (22.47 to 78.35) | 151 (62 to 232) | 2.52 (1.03 to 3.87) | 3,523 (1,426 to 5,401) | 61.88 (24.99 to 94.62) | 2.12 (1.63 to 2.60) | 1.62 (1.22 to 2.02) |
| Germany | 5,886 (2,454 to 8,839) | 4.47 (1.87 to 6.71) | 120,118 (50,937 to 179,774) | 96.14 (40.82 to 143.73) | 5,534 (2,213 to 8,482) | 2.62 (1.04 to 4.00) | 105,066 (41,404 to 161,403) | 57.81 (22.65 to 88.21) | -1.99 (-2.10 to -1.88) | -1.87 (-1.97 to -1.77) |
| Ghana | 52 (21 to 78) | 0.97 (0.40 to 1.43) | 1,439 (579 to 2,174) | 21.96 (9.01 to 32.79) | 178 (72 to 275) | 1.25 (0.51 to 1.92) | 4,697 (1,850 to 7,324) | 27.09 (10.87 to 41.9) | 1.04 (0.97 to 1.1) | 0.89 (0.83 to 0.96) |
| Greece | 452 (190 to 674) | 3.01 (1.27 to 4.5) | 9,254 (3,908 to 13,740) | 61.64 (26.07 to 91.61) | 736 (296 to 1,127) | 2.66 (1.07 to 4.05) | 12,970 (5,213 to 19,715) | 56.25 (22.63 to 84.88) | -0.75 (-0.95 to -0.54) | -0.55 (-0.71 to -0.39) |
| Greenland | 2 (1 to 3) | 7.59 (3.05 to 11.43) | 67 (27 to 99) | 179.12 (71.84 to 267.14) | 3 (1 to 4) | 4.4 (1.77 to 6.76) | 73 (30 to 112) | 102.19 (41.81 to 157.49) | -1.80 (-1.89 to -1.72) | -1.84 (-1.91 to -1.77) |
| Grenada | 2 (1 to 3) | 2.53 (1.04 to 3.82) | 42 (17 to 63) | 60.26 (24.84 to 91.08) | 3 (1 to 5) | 3.26 (1.32 to 5.08) | 83 (34 to 129) | 72.72 (29.66 to 112.9) | 1.06 (0.83 to 1.29) | 0.84 (0.69 to 0.98) |
| Guam | 2 (1 to 3) | 2.88 (1.20 to 4.35) | 52 (22 to 78) | 64.50 (27.08 to 96.03) | 4 (2 to 6) | 1.87 (0.74 to 2.85) | 108 (42 to 165) | 52.86 (20.71 to 80.49) | -0.76 (-1.13 to -0.39) | -0.11 (-0.42 to 0.20) |
| **Location** | **1990** | | | | **2021** | | | | **EAPC(1990-2021)** | |
|  | **Deaths Cases** | **ASMR**  **(/100k)** | **DALYs** | **ASDR**  **(/100k)** | **Deaths Cases** | **ASMR**  **(/100k)** | **DALYs** | **ASDR**  **(/100k)** | **ASMR** | **ASDR** |
|  | **No.**  **(95% UI)** | **No.**  **(95% UI)** | **No.**  **(95% UI)** | **No.**  **(95% UI)** | **No.**  **(95% UI)** | **No.**  **(95% UI)** | **No.**  **(95% UI)** | **No.**  **(95% UI)** | **No.**  **(95% CI)** | **No.**  **(95% CI)** |
| Guatemala | 24 (10 to 35) | 0.81 (0.33 to 1.19) | 692 (288 to 1,012) | 18.39 (7.65 to 26.92) | 128 (52 to 199) | 1.21 (0.49 to 1.86) | 3,380 (1,381 to 5,307) | 29.17 (11.93 to 45.68) | 1.12 (0.77 to 1.46) | 1.40 (1.08 to 1.72) |
| Guinea | 22 (9 to 35) | 0.71 (0.30 to 1.15) | 572 (237 to 902) | 16.85 (7.02 to 26.76) | 42 (17 to 68) | 0.80 (0.33 to 1.30) | 1,122 (465 to 1,874) | 18.75 (7.72 to 30.85) | 0.5 (0.41 to 0.58) | 0.47 (0.38 to 0.56) |
| Guinea-Bissau | 4 (2 to 7) | 1.2 (0.48 to 1.87) | 125 (49 to 198) | 29.35 (11.62 to 46.27) | 9 (4 to 14) | 1.40 (0.59 to 2.20) | 263 (107 to 426) | 32.52 (13.54 to 51.89) | 0.74 (0.66 to 0.83) | 0.56 (0.48 to 0.64) |
| Guyana | 7 (3 to 11) | 1.98 (0.83 to 3.04) | 189 (79 to 293) | 47.28 (19.70 to 72.69) | 14 (5 to 23) | 2.33 (0.89 to 3.70) | 386 (146 to 624) | 57.18 (21.72 to 92.26) | 0.78 (0.51 to 1.05) | 0.90 (0.64 to 1.17) |
| Haiti | 80 (33 to 132) | 2.77 (1.14 to 4.51) | 2,236 (915 to 3,756) | 65.72 (27.27 to 108.57) | 164 (70 to 277) | 2.53 (1.08 to 4.25) | 4,592 (1,970 to 7,754) | 59.12 (25.39 to 100.00) | -0.13 (-0.19 to -0.07) | -0.16 (-0.23 to -0.1) |
| Honduras | 14 (6 to 22) | 0.7 (0.29 to 1.07) | 413 (172 to 641) | 18.04 (7.50 to 27.73) | 64 (25 to 105) | 1.07 (0.42 to 1.75) | 1,689 (646 to 2,779) | 25.30 (9.82 to 41.47) | 1.53 (1.35 to 1.72) | 1.22 (1.07 to 1.37) |
| Hungary | 825 (325 to 1,295) | 5.67 (2.22 to 8.88) | 18,678 (7,378 to 29,120) | 128.68 (50.62 to 200.1) | 967 (406 to 1,470) | 4.82 (2.02 to 7.35) | 20,978 (8,797 to 32,038) | 113.76 (47.61 to 173.37) | -0.61 (-0.85 to -0.38) | -0.51 (-0.74 to -0.27) |
| **Location** | **1990** | | | | **2021** | | | | **EAPC(1990-2021)** | |
|  | **Deaths Cases** | **ASMR**  **(/100k)** | **DALYs** | **ASDR**  **(/100k)** | **Deaths Cases** | **ASMR**  **(/100k)** | **DALYs** | **ASDR**  **(/100k)** | **ASMR** | **ASDR** |
|  | **No.**  **(95% UI)** | **No.**  **(95% UI)** | **No.**  **(95% UI)** | **No.**  **(95% UI)** | **No.**  **(95% UI)** | **No.**  **(95% UI)** | **No.**  **(95% UI)** | **No.**  **(95% UI)** | **No.**  **(95% CI)** | **No.**  **(95% CI)** |
| Iceland | 9 (4 to 13) | 2.99 (1.25 to 4.56) | 187 (79 to 282) | 66.46 (28.06 to 100.41) | 13 (5 to 20) | 2.08 (0.84 to 3.17) | 253 (104 to 384) | 44.45 (18.38 to 67.36) | -1.08 (-1.21 to -0.96) | -1.27 (-1.37 to -1.16) |
| India | 3,388 (1,470 to 5,261) | 0.73 (0.32 to 1.14) | 105,823 (45,585 to 163,555) | 19.89 (8.61 to 30.83) | 9,140 (3,783 to 13,783) | 0.78 (0.32 to 1.17) | 258,515 (106,873 to 386,358) | 20.35 (8.42 to 30.46) | 0.11 (-0.02 to 0.24) | -0.04 (-0.17 to 0.09) |
| Indonesia | 1,340 (543 to 2,120) | 1.42 (0.59 to 2.24) | 40,691 (16,454 to 65,126) | 36.62 (14.84 to 58.09) | 4,197 (1,715 to 6,660) | 1.91 (0.77 to 3.02) | 118,228 (49,062 to 188,966) | 45.78 (18.81 to 72.72) | 0.99 (0.88 to 1.10) | 0.75 (0.64 to 0.86) |
| Iran (Islamic Republic of) | 318 (125 to 493) | 1.37 (0.54 to 2.12) | 9,348 (3,622 to 14,509) | 33.15 (12.98 to 51.55) | 1,022 (429 to 1,529) | 1.39 (0.59 to 2.08) | 26,723 (11,147 to 40,072) | 32.60 (13.60 to 48.88) | 0.52 (0.31 to 0.73) | 0.42 (0.20 to 0.64) |
| Iraq | 79 (32 to 118) | 0.99 (0.40 to 1.48) | 2,301 (928 to 3,502) | 26.52 (10.79 to 39.84) | 266 (102 to 429) | 1.19 (0.46 to 1.93) | 7,676 (2,975 to 12,425) | 28.98 (11.16 to 46.66) | 0.63 (0.42 to 0.84) | 0.34 (0.19 to 0.50) |
| Ireland | 182 (75 to 275) | 4.49 (1.86 to 6.81) | 3,927 (1,622 to 5,931) | 98.28 (40.52 to 148.16) | 200 (83 to 307) | 2.43 (1.02 to 3.73) | 4,049 (1,746 to 6,219) | 52.02 (22.41 to 79.77) | -1.74 (-1.85 to -1.63) | -1.85 (-1.96 to -1.75) |
| Israel | 194 (78 to 296) | 4.10 (1.66 to 6.29) | 4,074 (1,645 to 6,250) | 85.18 (34.44 to 130.75) | 333 (140 to 507) | 2.53 (1.05 to 3.84) | 6,231 (2,601 to 9,555) | 51.11 (21.61 to 78.17) | -2.14 (-2.44 to -1.83) | -2.12 (-2.42 to -1.83) |
| **Location** | **1990** | | | | **2021** | | | | **EAPC(1990-2021)** | |
|  | **Deaths Cases** | **ASMR**  **(/100k)** | **DALYs** | **ASDR**  **(/100k)** | **Deaths Cases** | **ASMR**  **(/100k)** | **DALYs** | **ASDR**  **(/100k)** | **ASMR** | **ASDR** |
|  | **No.**  **(95% UI)** | **No.**  **(95% UI)** | **No.**  **(95% UI)** | **No.**  **(95% UI)** | **No.**  **(95% UI)** | **No.**  **(95% UI)** | **No.**  **(95% UI)** | **No.**  **(95% UI)** | **No.**  **(95% CI)** | **No.**  **(95% CI)** |
| Italy | 3,374 (1,402 to 4,955) | 3.76 (1.56 to 5.52) | 72,462 (29,831 to 106,501) | 83.71 (34.39 to 123.12) | 4,450 (1,835 to 6,718) | 2.71 (1.12 to 4.07) | 81,194 (33,298 to 121,244) | 58.62 (24.14 to 87.05) | -1.10 (-1.22 to -0.99) | -1.21 (-1.35 to -1.07) |
| Jamaica | 37 (15 to 56) | 2.04 (0.82 to 3.05) | 788 (316 to 1,178) | 44.43 (17.84 to 66.33) | 88 (32 to 138) | 2.81 (1.03 to 4.39) | 2,025 (734 to 3,159) | 65.58 (23.74 to 102.39) | 1.10 (0.77 to 1.44) | 1.29 (0.91 to 1.68) |
| Japan | 5,201 (2,187 to 7,820) | 3.15 (1.33 to 4.75) | 124,644 (51,886 to 186,909) | 73.75 (30.74 to 110.57) | 11,887 (4,996 to 18,189) | 2.79 (1.17 to 4.23) | 206,061 (86,284 to 311,992) | 63.09 (26.59 to 93.86) | -0.41 (-0.46 to -0.36) | -0.53 (-0.59 to -0.48) |
| Jordan | 26 (11 to 40) | 2.06 (0.87 to 3.13) | 785 (332 to 1,214) | 51.52 (22.14 to 79.31) | 112 (45 to 182) | 1.66 (0.68 to 2.66) | 3,199 (1,302 to 5,214) | 38.8 (15.74 to 62.74) | -0.60 (-0.81 to -0.40) | -0.90 (-1.12 to -0.68) |
| Kazakhstan | 363 (155 to 550) | 2.91 (1.24 to 4.41) | 10,235 (4,353 to 15,444) | 76.65 (32.61 to 115.68) | 344 (147 to 535) | 1.99 (0.85 to 3.10) | 9,238 (3,944 to 14,243) | 49.12 (20.96 to 75.75) | -0.91 (-1.15 to -0.66) | -1.18 (-1.38 to -0.98) |
| Kenya | 63 (26 to 103) | 0.80 (0.33 to 1.29) | 1,817 (757 to 2,944) | 20.10 (8.39 to 32.67) | 261 (116 to 399) | 1.24 (0.53 to 1.90) | 7,443 (3,319 to 11,403) | 29.45 (13.11 to 45.07) | 1.92 (1.68 to 2.15) | 1.66 (1.43 to 1.89) |
| Kiribati | 1 (0 to 1) | 1.65 (0.64 to 2.64) | 17 (7 to 27) | 41.70 (16.06 to 66.02) | 1 (0 to 2) | 1.66 (0.74 to 2.70) | 33 (14 to 55) | 40.97 (17.82 to 67.19) | -0.09 (-0.17 to -0.01) | -0.18 (-0.25 to -0.11) |
| **Location** | **1990** | | | | **2021** | | | | **EAPC(1990-2021)** | |
|  | **Deaths Cases** | **ASMR**  **(/100k)** | **DALYs** | **ASDR**  **(/100k)** | **Deaths Cases** | **ASMR**  **(/100k)** | **DALYs** | **ASDR**  **(/100k)** | **ASMR** | **ASDR** |
|  | **No.**  **(95% UI)** | **No.**  **(95% UI)** | **No.**  **(95% UI)** | **No.**  **(95% UI)** | **No.**  **(95% UI)** | **No.**  **(95% UI)** | **No.**  **(95% UI)** | **No.**  **(95% UI)** | **No.**  **(95% CI)** | **No.**  **(95% CI)** |
| Kuwait | 6 (3 to 9) | 1.12 (0.47 to 1.72) | 182 (77 to 278) | 25.91 (10.91 to 39.44) | 43 (17 to 67) | 1.61 (0.65 to 2.5) | 1,242 (500 to 1,921) | 35.96 (14.46 to 55.89) | 1.62 (1.16 to 2.09) | 1.39 (0.9 to 1.88) |
| Kyrgyzstan | 61 (26 to 93) | 2.06 (0.87 to 3.14) | 1,725 (729 to 2,610) | 55.34 (23.34 to 83.92) | 62 (25 to 95) | 1.33 (0.54 to 2.05) | 1,794 (746 to 2,757) | 34.06 (14.11 to 52.68) | -1.17 (-1.37 to -0.97) | -1.41 (-1.56 to -1.26) |
| Lao People's Democratic Republic | 40 (15 to 67) | 2.01 (0.77 to 3.34) | 1,205 (438 to 2,027) | 52.77 (19.7 to 88.12) | 83 (32 to 139) | 1.90 (0.74 to 3.17) | 2,418 (929 to 4,032) | 47.02 (18.14 to 78.03) | -0.25 (-0.28 to -0.22) | -0.47 (-0.50 to -0.43) |
| Latvia | 121 (49 to 184) | 3.36 (1.35 to 5.09) | 2,899 (1,158 to 4,403) | 81.52 (32.52 to 123.59) | 132 (51 to 206) | 3.12 (1.21 to 4.88) | 2,659 (1,033 to 4,148) | 70.86 (27.53 to 110.04) | -0.17 (-0.33 to 0) | -0.48 (-0.63 to -0.33) |
| Lebanon | 51 (21 to 79) | 2.56 (1.04 to 4.00) | 1,288 (532 to 1,994) | 58.43 (23.99 to 90.37) | 132 (55 to 204) | 2.08 (0.87 to 3.22) | 2,701 (1,128 to 4,160) | 44.61 (18.65 to 68.87) | -0.20 (-0.44 to 0.03) | -0.42 (-0.64 to -0.21) |
| Lesotho | 9 (4 to 14) | 1.09 (0.46 to 1.80) | 214 (87 to 350) | 24.89 (10.24 to 40.98) | 20 (8 to 34) | 2.02 (0.80 to 3.39) | 565 (226 to 949) | 49.23 (19.43 to 82.7) | 2.77 (2.37 to 3.17) | 3.00 (2.56 to 3.44) |
| Liberia | 9 (4 to 15) | 0.88 (0.38 to 1.45) | 231 (102 to 385) | 19.71 (8.64 to 32.62) | 18 (7 to 34) | 0.99 (0.39 to 1.81) | 505 (195 to 938) | 21.84 (8.49 to 40.34) | 0.66 (0.48 to 0.83) | 0.58 (0.40 to 0.76) |
| **Location** | **1990** | | | | **2021** | | | | **EAPC(1990-2021)** | |
|  | **Deaths Cases** | **ASMR**  **(/100k)** | **DALYs** | **ASDR**  **(/100k)** | **Deaths Cases** | **ASMR**  **(/100k)** | **DALYs** | **ASDR**  **(/100k)** | **ASMR** | **ASDR** |
|  | **No.**  **(95% UI)** | **No.**  **(95% UI)** | **No.**  **(95% UI)** | **No.**  **(95% UI)** | **No.**  **(95% UI)** | **No.**  **(95% UI)** | **No.**  **(95% UI)** | **No.**  **(95% UI)** | **No.**  **(95% CI)** | **No.**  **(95% CI)** |
| Libya | 46 (18 to 71) | 2.56 (1.05 to 3.97) | 1,215 (487 to 1,892) | 60.77 (24.57 to 94.53) | 130 (54 to 211) | 2.64 (1.11 to 4.28) | 3,636 (1,525 to 5,985) | 62.60 (26.20 to 101.65) | 0.5 (0.32 to 0.68) | 0.42 (0.26 to 0.57) |
| Lithuania | 141 (59 to 211) | 3.12 (1.32 to 4.68) | 3,370 (1,425 to 5,040) | 75.19 (31.79 to 112.67) | 192 (76 to 295) | 3.12 (1.23 to 4.80) | 3,831 (1,504 to 5,868) | 69.91 (27.44 to 107.22) | -0.04 (-0.18 to 0.11) | -0.31 (-0.47 to -0.15) |
| Luxembourg | 26 (11 to 40) | 4.86 (2.03 to 7.29) | 564 (236 to 844) | 105.47 (44.19 to 157.67) | 30 (12 to 45) | 2.64 (1.06 to 3.96) | 576 (231 to 867) | 54.16 (21.68 to 81.45) | -1.91 (-2.06 to -1.76) | -2.08 -2.25 to -1.92) |
| Madagascar | 64 (25 to 103) | 1.34 (0.53 to 2.15) | 1,824 (715 to 2,908) | 33.21 (12.97 to 53.18) | 132 (50 to 217) | 1.32 (0.5 to 2.15) | 4,014 (1,505 to 6,668) | 31.32 (11.81 to 51.37) | 0.05 (-0.06 to 0.15) | -0.13 (-0.24 to -0.02) |
| Malawi | 22 (9 to 34) | 0.63 (0.26 to 0.97) | 629 (259 to 984) | 15.14 (6.29 to 23.68) | 49 (19 to 79) | 0.71 (0.28 to 1.13) | 1,403 (554 to 2,326) | 17.12 (6.79 to 27.77) | 0.26 (0.09 to 0.43) | 0.21 (0.01 to 0.40) |
| Malaysia | 259 (112 to 401) | 2.93 (1.27 to 4.53) | 6,967 (2,962 to 10,863) | 70.69 (30.42 to 109.87) | 849 (371 to 1,298) | 3.2 (1.39 to 4.91) | 21,682 (9,566 to 33,146) | 74.26 (32.69 to 113.82) | 0.10 (-0.03 to 0.23) | 0 (-0.15 to 0.15) |
| Maldives | 1 (0 to 2) | 1.37 (0.57 to 2.20) | 33 (13 to 55) | 33.73 (14.01 to 54.83) | 3 (1 to 4) | 0.81 (0.33 to 1.25) | 66 (27 to 102) | 17.35 (6.98 to 26.77) | -2.03 (-2.14 to -1.91) | -2.52 (-2.65 to -2.38) |
| **Location** | **1990** | | | | **2021** | | | | **EAPC(1990-2021)** | |
|  | **Deaths Cases** | **ASMR**  **(/100k)** | **DALYs** | **ASDR**  **(/100k)** | **Deaths Cases** | **ASMR**  **(/100k)** | **DALYs** | **ASDR**  **(/100k)** | **ASMR** | **ASDR** |
|  | **No.**  **(95% UI)** | **No.**  **(95% UI)** | **No.**  **(95% UI)** | **No.**  **(95% UI)** | **No.**  **(95% UI)** | **No.**  **(95% UI)** | **No.**  **(95% UI)** | **No.**  **(95% UI)** | **No.**  **(95% CI)** | **No.**  **(95% CI)** |
| Mali | 47 (20 to 70) | 1.30 (0.55 to 1.94) | 1,291 (543 to 1,921) | 30.84 (12.97 to 46) | 100 (41 to 155) | 1.27 (0.54 to 1.96) | 2,733 (1,111 to 4,224) | 29.16 (12.06 to 44.95) | 0.12 (0.01 to 0.22) | -0.01 (-0.12 to 0.1) |
| Malta | 15 (6 to 22) | 3.58 (1.50 to 5.32) | 330 (138 to 486) | 77.51 (32.44 to 114.42) | 27 (10 to 40) | 2.58 (0.99 to 3.85) | 519 (204 to 774) | 55.22 (21.80 to 82.19) | -1.11 (-1.22 to -1.01) | -1.13 (-1.22 to -1.04) |
| Marshall Islands | 0 (0 to 1) | 2.16 (0.90 to 3.41) | 9 (4 to 15) | 53.07 (21.87 to 83.98) | 1 (0 to 1) | 2.22 (0.93 to 3.57) | 22 (9 to 36) | 55.02 (23.53 to 89.78) | 0.10 (0.04 to 0.17) | 0.13 (0.05 to 0.20) |
| Mauritania | 11 (4 to 17) | 1.21 (0.49 to 1.84) | 280 (113 to 437) | 27.59 (11.08 to 42.76) | 26 (11 to 42) | 1.33 (0.55 to 2.15) | 620 (256 to 1,021) | 28.41 (11.73 to 46.68) | 0.35 (0.19 to 0.52) | 0.11 (-0.05 to 0.28) |
| Mauritius | 10 (4 to 15) | 1.50 (0.64 to 2.25) | 280 (119 to 416) | 36.25 (15.41 to 54.05) | 46 (19 to 69) | 2.58 (1.07 to 3.84) | 1,162 (480 to 1,740) | 63.77 (26.34 to 95.52) | 1.19 (1.01 to 1.38) | 1.25 (1.05 to 1.45) |
| Mexico | 389 (160 to 583) | 1.01 (0.41 to 1.52) | 9,984 (4,139 to 14,941) | 22.21 (9.18 to 33.28) | 1,723 (710 to 2,665) | 1.38 (0.57 to 2.14) | 46,027 (19,092 to 71,293) | 35.07 (14.53 to 54.28) | 1.15 (1.00 to 1.31) | 1.58 (1.45 to 1.72) |
| Micronesia (Federated States of) | 1 (0 to 2) | 2.37 (0.97 to 3.70) | 30 (12 to 47) | 58.38 (23.52 to 91.81) | 2 (1 to 3) | 2.30 (1.00 to 3.80) | 45 (20 to 74) | 55.85 (24.87 to 92.28) | -0.11 (-0.14 to -0.07) | -0.14 (-0.18 to -0.10) |
| **Location** | **1990** | | | | **2021** | | | | **EAPC(1990-2021)** | |
|  | **Deaths Cases** | **ASMR**  **(/100k)** | **DALYs** | **ASDR**  **(/100k)** | **Deaths Cases** | **ASMR**  **(/100k)** | **DALYs** | **ASDR**  **(/100k)** | **ASMR** | **ASDR** |
|  | **No.**  **(95% UI)** | **No.**  **(95% UI)** | **No.**  **(95% UI)** | **No.**  **(95% UI)** | **No.**  **(95% UI)** | **No.**  **(95% UI)** | **No.**  **(95% UI)** | **No.**  **(95% UI)** | **No.**  **(95% CI)** | **No.**  **(95% CI)** |
| Monaco | 3 (1 to 5) | 4.47 (1.87 to 6.98) | 65 (27 to 102) | 98.72 (40.97 to 154.6) | 5 (2 to 8) | 4.68 (1.8 to 7.11) | 93 (36 to 142) | 102 (39.06 to 156.31) | 0.26 (0.16 to 0.36) | 0.22 (0.12 to 0.33) |
| Mongolia | 15 (6 to 24) | 1.45 (0.55 to 2.27) | 440 (168 to 687) | 38.77 (14.66 to 60.65) | 34 (13 to 52) | 1.53 (0.61 to 2.33) | 1,011 (400 to 1,544) | 38.86 (15.4 to 59.31) | 0.01 (-0.12 to 0.14) | -0.16 (-0.30 to -0.03) |
| Montenegro | 16 (7 to 25) | 2.62 (1.11 to 4.05) | 393 (166 to 611) | 61.96 (26.21 to 96.23) | 32 (13 to 48) | 3.33 (1.38 to 5.07) | 688 (293 to 1,042) | 70.91 (30.01 to 107.93) | 0.90 (0.78 to 1.03) | 0.57 (0.47 to 0.67) |
| Morocco | 155 (66 to 242) | 1.13 (0.48 to 1.77) | 4,230 (1,784 to 6,592) | 28 (11.84 to 43.81) | 516 (225 to 806) | 1.58 (0.68 to 2.46) | 13,545 (5,876 to 21,527) | 37.88 (16.45 to 59.76) | 1.33 (1.22 to 1.43) | 1.18 (1.09 to 1.28) |
| Mozambique | 29 (12 to 45) | 0.60 (0.25 to 0.94) | 718 (292 to 1,121) | 12.49 (5.06 to 19.48) | 66 (27 to 106) | 0.75 (0.30 to 1.19) | 1,708 (700 to 2,723) | 15.64 (6.3 to 24.86) | 1.17 (1.02 to 1.32) | 1.20 (1.04 to 1.37) |
| Myanmar | 427 (179 to 726) | 1.90 (0.81 to 3.24) | 12,777 (5,315 to 21,885) | 50.09 (20.9 to 85.14) | 870 (337 to 1,403) | 1.87 (0.74 to 3.00) | 23,686 (8,963 to 37,858) | 46.17 (17.56 to 74.16) | -0.14 (-0.2 to -0.08) | -0.37 (-0.45 to -0.30) |
| Namibia | 6 (3 to 10) | 1.04 (0.43 to 1.65) | 179 (75 to 281) | 25.78 (10.76 to 40.64) | 15 (6 to 23) | 1.12 (0.46 to 1.79) | 418 (172 to 671) | 27.62 (11.43 to 43.90) | 0.16 (-0.07 to 0.38) | 0.08 (-0.18 to 0.34) |
| **Location** | **1990** | | | | **2021** | | | | **EAPC(1990-2021)** | |
|  | **Deaths Cases** | **ASMR**  **(/100k)** | **DALYs** | **ASDR**  **(/100k)** | **Deaths Cases** | **ASMR**  **(/100k)** | **DALYs** | **ASDR**  **(/100k)** | **ASMR** | **ASDR** |
|  | **No.**  **(95% UI)** | **No.**  **(95% UI)** | **No.**  **(95% UI)** | **No.**  **(95% UI)** | **No.**  **(95% UI)** | **No.**  **(95% UI)** | **No.**  **(95% UI)** | **No.**  **(95% UI)** | **No.**  **(95% CI)** | **No.**  **(95% CI)** |
| Nauru | 0 (0 to 0) | 3.39 (1.18 to 5.78) | 4 (2 to 8) | 85.00 (30.13 to 147.49) | 0 (0 to 0) | 3.12 (1.21 to 4.99) | 5 (2 to 8) | 78.33 (31.22 to 126.79) | -0.32 (-0.36 to -0.28) | -0.33 (-0.36 to -0.29) |
| Nepal | 62 (25 to 104) | 0.68 (0.28 to 1.12) | 1,872 (736 to 3,216) | 17.69 (7.09 to 30.09) | 153 (65 to 254) | 0.69 (0.29 to 1.14) | 4,211 (1,750 to 6,840) | 17.18 (7.19 to 28.07) | 0.18 (-0.15 to 0.51) | 0.01 (-0.32 to 0.35) |
| Netherlands | 881 (361 to 1,327) | 4.33 (1.77 to 6.51) | 18,928 (7,719 to 27,967) | 96.2 (39.41 to 141.79) | 1,373 (540 to 2,060) | 3.68 (1.45 to 5.53) | 27,924 (11,150 to 41,921) | 81.70 (32.99 to 122.79) | -0.43 (-0.59 to -0.27) | -0.44 (-0.60 to -0.28) |
| New Zealand | 205 (88 to 310) | 5.29 (2.27 to 8.00) | 4,730 (2,023 to 7,189) | 123.41 (52.70 to 187.57) | 290 (122 to 445) | 3.29 (1.38 to 5.05) | 5,795 (2,498 to 8,832) | 70.78 (30.73 to 107.68) | -1.60 (-1.67 to -1.53) | -1.93 (-2.02 to -1.85) |
| Nicaragua | 12 (5 to 18) | 0.80 (0.33 to 1.21) | 329 (136 to 496) | 19.36 (7.99 to 29.2) | 47 (19 to 74) | 0.99 (0.40 to 1.57) | 1,266 (517 to 1,985) | 24.38 (9.96 to 38.24) | 1.12 (0.86 to 1.39) | 1.12 (0.89 to 1.36) |
| Niger | 20 (9 to 33) | 0.83 (0.35 to 1.35) | 561 (235 to 922) | 19.11 (8.15 to 31.43) | 63 (25 to 105) | 0.91 (0.37 to 1.52) | 1,671 (661 to 2,735) | 19.81 (8.02 to 32.68) | 0.54 (0.44 to 0.64) | 0.31 (0.21 to 0.41) |
| Nigeria | 384 (155 to 613) | 0.95 (0.39 to 1.51) | 9,886 (3,991 to 15,940) | 21.72 (8.76 to 34.96) | 824 (340 to 1,272) | 1.04 (0.44 to 1.58) | 21,667 (8,678 to 34,087) | 22.57 (9.26 to 34.96) | 0.44 (0.37 to 0.51) | 0.26 (0.19 to 0.33) |
| **Location** | **1990** | | | | **2021** | | | | **EAPC(1990-2021)** | |
|  | **Deaths Cases** | **ASMR**  **(/100k)** | **DALYs** | **ASDR**  **(/100k)** | **Deaths Cases** | **ASMR**  **(/100k)** | **DALYs** | **ASDR**  **(/100k)** | **ASMR** | **ASDR** |
|  | **No.**  **(95% UI)** | **No.**  **(95% UI)** | **No.**  **(95% UI)** | **No.**  **(95% UI)** | **No.**  **(95% UI)** | **No.**  **(95% UI)** | **No.**  **(95% UI)** | **No.**  **(95% UI)** | **No.**  **(95% CI)** | **No.**  **(95% CI)** |
| Niue | 0 (0 to 0) | 2.11 (0.89 to 3.22) | 1 (0 to 2) | 49.42 (21.07 to 75.23) | 0 (0 to 0) | 2.21 (0.86 to 3.43) | 1 (0 to 2) | 51.27 (19.71 to 81.53) | 0.07 (0.03 to 0.10) | -0.02 (-0.06 to 0.02) |
| North Macedonia | 54 (23 to 81) | 3.03 (1.28 to 4.55) | 1,343 (568 to 2,029) | 70.54 (29.72 to 105.76) | 111 (45 to 170) | 3.64 (1.46 to 5.55) | 2,520 (1,009 to 3,865) | 76.31 (30.56 to 116.66) | 0.61 (0.27 to 0.96) | 0.29 (0 to 0.59) |
| Northern Mariana Islands | 0 (0 to 1) | 2.86 (1.18 to 4.36) | 15 (6 to 23) | 66.74 (27.45 to 103.02) | 1 (1 to 2) | 2.86 (1.15 to 4.29) | 36 (14 to 54) | 65.78 (26.59 to 99.00) | 0.03 (-0.19 to 0.26) | 0.01 (-0.22 to 0.25) |
| Norway | 323 (137 to 489) | 4.51 (1.91 to 6.82) | 6,463 (2,713 to 9,747) | 99.44 (41.70 to 149.72) | 373 (156 to 558) | 3.39 (1.42 to 5.08) | 6,854 (2,869 to 10,192) | 69.04 (28.79 to 102.59) | -0.99 (-1.07 to -0.90) | -1.23 (-1.31 to -1.14) |
| Oman | 5 (2 to 8) | 0.79 (0.31 to 1.25) | 143 (57 to 229) | 19.02 (7.49 to 30.40) | 11 (5 to 19) | 0.68 (0.29 to 1.08) | 336 (136 to 548) | 14.93 (6.09 to 24.23) | -0.07 (-0.29 to 0.14) | -0.42 (-0.67 to -0.17) |
| Pakistan | 508 (217 to 775) | 0.95 (0.41 to 1.46) | 13,869 (5,934 to 21,228) | 23.37 (10.00 to 35.74) | 1,355 (546 to 2,156) | 1.19 (0.48 to 1.87) | 39,268 (15,678 to 62,383) | 28.84 (11.59 to 45.99) | 0.46 (0.24 to 0.68) | 0.39 (0.16 to 0.62) |
| Palau | 0 (0 to 0) | 2.78 (1.11 to 4.31) | 6 (2 to 10) | 62.95 (24.77 to 98.5) | 0 (0 to 1) | 2.48 (1.00 to 3.91) | 12 (5 to 18) | 52.76 (21.20 to 82.99) | -0.20 (-0.30 to -0.10) | -0.44 (-0.53 to -0.35) |
| **Location** | **1990** | | | | **2021** | | | | **EAPC(1990-2021)** | |
|  | **Deaths Cases** | **ASMR**  **(/100k)** | **DALYs** | **ASDR**  **(/100k)** | **Deaths Cases** | **ASMR**  **(/100k)** | **DALYs** | **ASDR**  **(/100k)** | **ASMR** | **ASDR** |
|  | **No.**  **(95% UI)** | **No.**  **(95% UI)** | **No.**  **(95% UI)** | **No.**  **(95% UI)** | **No.**  **(95% UI)** | **No.**  **(95% UI)** | **No.**  **(95% UI)** | **No.**  **(95% UI)** | **No.**  **(95% CI)** | **No.**  **(95% CI)** |
| Palestine | 28 (11 to 45) | 3.46 (1.40 to 5.60) | 716 (280 to 1,170) | 79.89 (31.47 to 130.13) | 64 (27 to 98) | 2.86 (1.20 to 4.32) | 1,743 (731 to 2,671) | 64.17 (26.94 to 98.13) | -0.55 (-0.72 to -0.39) | -0.66 (-0.78 to -0.53) |
| Panama | 21 (9 to 32) | 1.48 (0.61 to 2.21) | 535 (224 to 795) | 34.59 (14.46 to 51.53) | 80 (29 to 125) | 1.79 (0.65 to 2.81) | 1,925 (708 to 3,039) | 43.58 (16.03 to 68.82) | 0.82 (0.74 to 0.90) | 0.96 (0.87 to 1.05) |
| Papua New Guinea | 12 (5 to 20) | 0.72 (0.29 to 1.15) | 400 (153 to 664) | 18.79 (7.4 to 30.68) | 32 (12 to 50) | 0.66 (0.26 to 1.03) | 1,031 (395 to 1,616) | 16.88 (6.54 to 26.29) | -0.35 (-0.42 to -0.27) | -0.43 (-0.52 to -0.35) |
| Paraguay | 23 (10 to 36) | 1.09 (0.45 to 1.67) | 583 (235 to 908) | 25.19 (10.19 to 39.15) | 103 (38 to 162) | 1.83 (0.68 to 2.89) | 2,521 (931 to 3,980) | 42.21 (15.50 to 66.6) | 2.05 (1.89 to 2.21) | 1.99 (1.82 to 2.15) |
| Peru | 172 (72 to 268) | 1.51 (0.63 to 2.34) | 4,316 (1,771 to 6,767) | 34.57 (14.23 to 53.75) | 541 (202 to 875) | 1.63 (0.61 to 2.63) | 12,427 (4,621 to 20,198) | 36.58 (13.61 to 59.33) | 0.15 (-0.03 to 0.34) | 0.08 (-0.10 to 0.27) |
| Philippines | 485 (199 to 737) | 1.72 (0.70 to 2.62) | 15,089 (6,096 to 22,871) | 43.66 (17.91 to 66.44) | 1,693 (696 to 2,594) | 2.11 (0.87 to 3.24) | 49,410 (20,161 to 75,814) | 54.60 (22.40 to 83.45) | 0.87 (0.81 to 0.93) | 0.84 (0.79 to 0.89) |
| Poland | 1,760 (742 to 2,608) | 4.08 (1.72 to 6.05) | 41,352 (17,423 to 61,116) | 94.53 (39.81 to 139.78) | 3,354 (1,420 to 5,021) | 4.49 (1.90 to 6.71) | 67,508 (28,675 to 100,707) | 95.96 (40.87 to 142.96) | 0.09 (-0.07 to 0.24) | -0.08 (-0.23 to 0.07) |
| **Location** | **1990** | | | | **2021** | | | | **EAPC(1990-2021)** | |
|  | **Deaths Cases** | **ASMR**  **(/100k)** | **DALYs** | **ASDR**  **(/100k)** | **Deaths Cases** | **ASMR**  **(/100k)** | **DALYs** | **ASDR**  **(/100k)** | **ASMR** | **ASDR** |
|  | **No.**  **(95% UI)** | **No.**  **(95% UI)** | **No.**  **(95% UI)** | **No.**  **(95% UI)** | **No.**  **(95% UI)** | **No.**  **(95% UI)** | **No.**  **(95% UI)** | **No.**  **(95% UI)** | **No.**  **(95% CI)** | **No.**  **(95% CI)** |
| Portugal | 574 (247 to 865) | 4.30 (1.85 to 6.49) | 12,380 (5,346 to 18,622) | 91.77 (39.66 to 138.14) | 868 (362 to 1,302) | 3.21 (1.33 to 4.77) | 16,280 (6,659 to 24,240) | 70.90 (29.22 to 106.30) | -0.86 (-1.03 to -0.68) | -0.73 (-0.95 to -0.52) |
| Puerto Rico | 94 (39 to 142) | 2.68 (1.12 to 4.03) | 2,207 (917 to 3,319) | 61.53 (25.61 to 92.47) | 186 (71 to 290) | 2.49 (0.94 to 3.88) | 3,906 (1,488 to 6,086) | 62.05 (23.74 to 96.18) | -0.27 (-0.47 to -0.07) | 0 (-0.17 to 0.17) |
| Qatar | 2 (1 to 4) | 2.76 (1.12 to 4.19) | 78 (31 to 117) | 57.95 (23.41 to 87.82) | 15 (6 to 24) | 2.15 (0.86 to 3.39) | 501 (205 to 808) | 43.65 (17.48 to 69.79) | -0.62 (-1.28 to 0.05) | -0.65 (-1.24 to -0.06) |
| Republic of Korea | 562 (234 to 864) | 2.12 (0.90 to 3.28) | 15,963 (6,607 to 24,597) | 49.69 (20.72 to 76.25) | 1,901 (784 to 3,035) | 2.05 (0.85 to 3.28) | 39,708 (16,356 to 63,427) | 43.30 (17.92 to 69.28) | -0.26 (-0.46 to -0.06) | -0.58 (-0.80 to -0.35) |
| Republic of Moldova | 142 (58 to 218) | 3.33 (1.38 to 5.09) | 3,785 (1,556 to 5,784) | 83.39 (34.31 to 127.6) | 198 (84 to 308) | 3.28 (1.39 to 5.11) | 4,866 (2,074 to 7,601) | 81.76 (34.8 to 127.75) | 0.40 (-0.04 to 0.84) | 0.38 (-0.02 to 0.78) |
| Romania | 643 (263 to 972) | 2.35 (0.96 to 3.55) | 16,962 (6,894 to 25,914) | 60.24 (24.50 to 91.74) | 1,456 (605 to 2,305) | 3.82 (1.58 to 6.04) | 32,123 (13,306 to 50,371) | 91.31 (37.68 to 143.95) | 1.30 (1.08 to 1.52) | 1.06 (0.84 to 1.28) |
| Russian Federation | 5,812 (2,400 to 8,549) | 3.26 (1.35 to 4.79) | 148,490 (61,248 to 218,852) | 81.30 (33.54 to 119.84) | 8,531 (3,506 to 12,623) | 3.53 (1.45 to 5.22) | 191,189 (78,837 to 282,241) | 80.84 (33.37 to 119.35) | 0.07 (-0.09 to 0.22) | -0.26 (-0.43 to -0.09) |
| **Location** | **1990** | | | | **2021** | | | | **EAPC(1990-2021)** | |
|  | **Deaths Cases** | **ASMR**  **(/100k)** | **DALYs** | **ASDR**  **(/100k)** | **Deaths Cases** | **ASMR**  **(/100k)** | **DALYs** | **ASDR**  **(/100k)** | **ASMR** | **ASDR** |
|  | **No.**  **(95% UI)** | **No.**  **(95% UI)** | **No.**  **(95% UI)** | **No.**  **(95% UI)** | **No.**  **(95% UI)** | **No.**  **(95% UI)** | **No.**  **(95% UI)** | **No.**  **(95% UI)** | **No.**  **(95% CI)** | **No.**  **(95% CI)** |
| Rwanda | 54 (22 to 87) | 2.02 (0.84 to 3.27) | 1,592 (664 to 2,578) | 51.17 (21.36 to 83.06) | 96 (39 to 158) | 1.74 (0.71 to 2.82) | 2,663 (1,069 to 4,411) | 39.40 (15.92 to 65.23) | -1.17 (-1.45 to -0.89) | -1.63 (-1.95 to -1.32) |
| Saint Kitts and Nevis | 1 (0 to 2) | 3.18 (1.33 to 4.8) | 26 (11 to 39) | 72.54 (30.18 to 109.08) | 2 (1 to 3) | 3.20 (1.29 to 4.96) | 48 (19 to 76) | 68.97 (27.35 to 107.69) | 0.54 (0.38 to 0.70) | 0.28 (0.13 to 0.44) |
| Saint Lucia | 2 (1 to 3) | 2.35 (0.96 to 3.58) | 44 (18 to 66) | 51.05 (21.08 to 77.00) | 5 (2 to 8) | 2.08 (0.82 to 3.34) | 109 (43 to 177) | 45.76 (18.00 to 73.88) | -0.79 (-1.01 to -0.57) | -0.57 (-0.74 to -0.4) |
| Saint Vincent and the Grenadines | 2 (1 to 2) | 2.21 (0.89 to 3.33) | 35 (14 to 52) | 48.79 (19.59 to 73.25) | 3 (1 to 5) | 2.46 (0.97 to 3.84) | 79 (31 to 123) | 55.83 (21.99 to 87.46) | 0.40 (0.26 to 0.54) | 0.43 (0.31 to 0.56) |
| Samoa | 1 (1 to 2) | 1.8 (0.72 to 2.76) | 36 (14 to 56) | 41.49 (16.46 to 64.31) | 2 (1 to 4) | 1.76 (0.75 to 2.72) | 62 (26 to 95) | 41.55 (17.60 to 63.33) | -0.08 (-0.15 to -0.02) | 0 (-0.08 to 0.08) |
| San Marino | 2 (1 to 2) | 4.44 (1.84 to 6.6) | 32 (13 to 47) | 91.11 (36.98 to 136.2) | 2 (1 to 3) | 2.08 (0.83 to 3.52) | 32 (13 to 55) | 44.59 (17.45 to 76.76) | -1.51 (-1.86 to -1.17) | -1.42 (-1.74 to -1.09) |
| Sao Tome and Principe | 1 (0 to 1) | 1.53 (0.63 to 2.39) | 22 (9 to 33) | 33.37 (13.54 to 51.67) | 2 (1 to 3) | 2.03 (0.86 to 3.20) | 48 (19 to 78) | 42.88 (17.76 to 68.30) | 1.14 (1.07 to 1.20) | 0.94 (0.88 to 1.01) |
| **Location** | **1990** | | | | **2021** | | | | **EAPC(1990-2021)** | |
|  | **Deaths Cases** | **ASMR**  **(/100k)** | **DALYs** | **ASDR**  **(/100k)** | **Deaths Cases** | **ASMR**  **(/100k)** | **DALYs** | **ASDR**  **(/100k)** | **ASMR** | **ASDR** |
|  | **No.**  **(95% UI)** | **No.**  **(95% UI)** | **No.**  **(95% UI)** | **No.**  **(95% UI)** | **No.**  **(95% UI)** | **No.**  **(95% UI)** | **No.**  **(95% UI)** | **No.**  **(95% UI)** | **No.**  **(95% CI)** | **No.**  **(95% CI)** |
| Saudi Arabia | 61 (24 to 98) | 1.08 (0.42 to 1.71) | 1,844 (720 to 2,991) | 26.62 (10.33 to 42.82) | 270 (109 to 434) | 1.42 (0.58 to 2.26) | 9,323 (3,783 to 15,073) | 35.33 (14.30 to 56.24) | 1.08 (0.74 to 1.43) | 1.19 (0.86 to 1.52) |
| Senegal | 30 (13 to 45) | 1.02 (0.43 to 1.53) | 776 (323 to 1,169) | 23.30 (9.78 to 35.02) | 85 (36 to 136) | 1.23 (0.52 to 1.99) | 2,123 (912 to 3,480) | 26.74 (11.49 to 43.17) | 0.76 (0.62 to 0.90) | 0.59 (0.44 to 0.75) |
| Serbia | 462 (182 to 709) | 4.67 (1.83 to 7.10) | 11,412 (4,522 to 17,648) | 103.54 (40.89 to 158.84) | 678 (258 to 1,048) | 3.98 (1.51 to 6.15) | 14,560 (5,383 to 22,599) | 90.41 (33.25 to 140.84) | -0.58 (-0.67 to -0.49) | -0.52 (-0.64 to -0.40) |
| Seychelles | 2 (1 to 2) | 2.70 (1.08 to 4.15) | 38 (15 to 58) | 66.66 (26.64 to 103.06) | 4 (2 to 5) | 3.32 (1.42 to 4.99) | 94 (40 to 141) | 78.14 (33.58 to 117.22) | 0.81 (0.56 to 1.06) | 0.63 (0.40 to 0.87) |
| Sierra Leone | 15 (6 to 23) | 0.78 (0.30 to 1.20) | 365 (142 to 576) | 17.56 (6.78 to 27.43) | 31 (13 to 51) | 0.93 (0.37 to 1.50) | 818 (330 to 1,346) | 20.55 (8.22 to 33.61) | 0.88 (0.75 to 1.01) | 0.86 (0.72 to 1.00) |
| Singapore | 76 (31 to 115) | 3.68 (1.51 to 5.55) | 2,012 (824 to 3,033) | 86.04 (35.36 to 129.66) | 176 (71 to 269) | 2.08 (0.84 to 3.19) | 3,935 (1,584 to 6,039) | 45.66 (18.34 to 70.05) | -1.99 (-2.18 to -1.8) | -2.14 (-2.34 to -1.95) |
| Slovakia | 299 (122 to 451) | 4.99 (2.04 to 7.53) | 7,205 (2,928 to 10,873) | 120.45 (48.95 to 181.93) | 444 (172 to 687) | 4.59 (1.78 to 7.10) | 9,831 (3,851 to 15,366) | 104.28 (40.80 to 162.61) | -0.27 (-0.37 to -0.18) | -0.47 (-0.56 to -0.37) |
| **Location** | **1990** | | | | **2021** | | | | **EAPC(1990-2021)** | |
|  | **Deaths Cases** | **ASMR**  **(/100k)** | **DALYs** | **ASDR**  **(/100k)** | **Deaths Cases** | **ASMR**  **(/100k)** | **DALYs** | **ASDR**  **(/100k)** | **ASMR** | **ASDR** |
|  | **No.**  **(95% UI)** | **No.**  **(95% UI)** | **No.**  **(95% UI)** | **No.**  **(95% UI)** | **No.**  **(95% UI)** | **No.**  **(95% UI)** | **No.**  **(95% UI)** | **No.**  **(95% UI)** | **No.**  **(95% CI)** | **No.**  **(95% CI)** |
| Slovenia | 96 (40 to 145) | 3.87 (1.62 to 5.85) | 2,189 (917 to 3,320) | 88.75 (37.07 to 134.47) | 141 (57 to 222) | 2.91 (1.17 to 4.57) | 2,628 (1,053 to 4,133) | 60.48 (24.24 to 95.24) | -1.12 (-1.40 to -0.84) | -1.47 (-1.76 to -1.18) |
| Solomon Islands | 2 (1 to 4) | 1.76 (0.64 to 2.88) | 65 (22 to 112) | 43.37 (15.19 to 73.26) | 6 (2 to 9) | 1.77 (0.74 to 2.89) | 174 (72 to 293) | 44.07 (18.41 to 73.33) | 0.01 (-0.10 to 0.13) | 0.05 (-0.08 to 0.19) |
| Somalia | 40 (15 to 72) | 1.79 (0.7 to 3.17) | 1,282 (479 to 2,353) | 45.22 (17.36 to 80.8) | 101 (40 to 176) | 1.83 (0.76 to 3.2) | 3,159 (1,212 to 5,532) | 44.74 (17.76 to 77.41) | 0.23 (0.17 to 0.29) | 0.06 (0.01 to 0.11) |
| South Africa | 257 (108 to 405) | 1.31 (0.55 to 2.08) | 7,047 (2,977 to 10,891) | 31.26 (13.22 to 48.71) | 755 (317 to 1,144) | 1.75 (0.73 to 2.65) | 20,015 (8,436 to 30,216) | 41.04 (17.24 to 62.09) | 1.02 (0.77 to 1.28) | 1.07 (0.82 to 1.32) |
| South Sudan | 40 (15 to 68) | 1.68 (0.64 to 2.81) | 1,087 (418 to 1,833) | 40.64 (15.64 to 68.19) | 70 (29 to 112) | 2.00 (0.83 to 3.21) | 2,024 (827 to 3,282) | 47.31 (19.37 to 75.63) | 0.67 (0.57 to 0.77) | 0.54 (0.41 to 0.68) |
| Spain | 2,023 (858 to 3,048) | 3.71 (1.57 to 5.61) | 43,513 (18,247 to 65,478) | 82.27 (34.40 to 123.47) | 3,331 (1,419 to 4,965) | 3.04 (1.29 to 4.52) | 62,354 (26,372 to 92,407) | 66.06 (27.85 to 97.13) | -0.55 (-0.67 to -0.43) | -0.65 (-0.77 to -0.52) |
| Sri Lanka | 86 (36 to 129) | 0.87 (0.37 to 1.31) | 2,359 (997 to 3,541) | 20.50 (8.68 to 30.69) | 202 (70 to 335) | 0.77 (0.27 to 1.27) | 5,055 (1,778 to 8,460) | 18.58 (6.54 to 31.22) | -0.03 (-0.20 to 0.13) | -0.02 (-0.19 to 0.15) |
| **Location** | **1990** | | | | **2021** | | | | **EAPC(1990-2021)** | |
|  | **Deaths Cases** | **ASMR**  **(/100k)** | **DALYs** | **ASDR**  **(/100k)** | **Deaths Cases** | **ASMR**  **(/100k)** | **DALYs** | **ASDR**  **(/100k)** | **ASMR** | **ASDR** |
|  | **No.**  **(95% UI)** | **No.**  **(95% UI)** | **No.**  **(95% UI)** | **No.**  **(95% UI)** | **No.**  **(95% UI)** | **No.**  **(95% UI)** | **No.**  **(95% UI)** | **No.**  **(95% UI)** | **No.**  **(95% CI)** | **No.**  **(95% CI)** |
| Sudan | 108 (42 to 180) | 1.20 (0.47 to 1.98) | 3,114 (1,169 to 5,332) | 30.81 (11.71 to 51.56) | 249 (91 to 437) | 1.31 (0.49 to 2.29) | 7,356 (2,687 to 13,067) | 32.63 (11.96 to 57.44) | 0.38 (0.31 to 0.44) | 0.28 (0.22 to 0.34) |
| Suriname | 5 (2 to 8) | 2.21 (0.92 to 3.26) | 137 (57 to 204) | 52.10 (21.52 to 77.09) | 15 (6 to 24) | 2.43 (1.02 to 3.92) | 373 (157 to 595) | 58.05 (24.39 to 92.65) | 0.66 (0.45 to 0.86) | 0.62 (0.42 to 0.82) |
| Sweden | 557 (235 to 839) | 3.51 (1.49 to 5.28) | 11,017 (4,611 to 16,425) | 76.65 (32.12 to 113.5) | 652 (274 to 998) | 2.68 (1.13 to 4.12) | 11,487 (4,878 to 17,733) | 54.49 (23.38 to 83.45) | -0.77 (-0.93 to -0.62) | -0.90 (-1.07 to -0.74) |
| Switzerland | 303 (128 to 462) | 2.79 (1.18 to 4.24) | 6,288 (2,639 to 9,420) | 62.27 (26.11 to 92.85) | 393 (166 to 598) | 1.95 (0.83 to 2.94) | 7,302 (3,105 to 10,918) | 41.13 (17.75 to 61.17) | -1.26 (-1.43 to -1.1) | -1.46 (-1.64 to -1.28) |
| Syrian Arab Republic | 63 (25 to 99) | 1.25 (0.50 to 1.94) | 1,850 (741 to 2,933) | 31.53 (12.64 to 49.87) | 143 (58 to 236) | 1.21 (0.49 to 1.96) | 3,852 (1,581 to 6,519) | 28.25 (11.52 to 46.81) | -0.2 (-0.35 to -0.05) | -0.50 (-0.67 to -0.32) |
| Taiwan (Province of China) | 457 (198 to 684) | 3.06 (1.33 to 4.57) | 12,853 (5,544 to 19,270) | 75.82 (32.74 to 113.8) | 1,702 (669 to 2,584) | 3.98 (1.56 to 6.01) | 37,437 (14,819 to 56,716) | 91.28 (36.17 to 138.14) | 0.51 (0.19 to 0.84) | 0.27 (0 to 0.54) |
| Tajikistan | 40 (16 to 61) | 1.46 (0.58 to 2.24) | 1,173 (474 to 1,779) | 39.66 (16.02 to 60.74) | 50 (20 to 79) | 0.88 (0.36 to 1.40) | 1,539 (602 to 2,507) | 22.83 (9.03 to 36.41) | -1.57 (-1.78 to -1.35) | -1.81 (-2.01 to -1.61) |
| **Location** | **1990** | | | | **2021** | | | | **EAPC(1990-2021)** | |
|  | **Deaths Cases** | **ASMR**  **(/100k)** | **DALYs** | **ASDR**  **(/100k)** | **Deaths Cases** | **ASMR**  **(/100k)** | **DALYs** | **ASDR**  **(/100k)** | **ASMR** | **ASDR** |
|  | **No.**  **(95% UI)** | **No.**  **(95% UI)** | **No.**  **(95% UI)** | **No.**  **(95% UI)** | **No.**  **(95% UI)** | **No.**  **(95% UI)** | **No.**  **(95% UI)** | **No.**  **(95% UI)** | **No.**  **(95% CI)** | **No.**  **(95% CI)** |
| Thailand | 711 (318 to 1,135) | 2.15 (0.96 to 3.43) | 19,899 (8,891 to 31,580) | 51.81 (23.14 to 82.58) | 2,519 (955 to 4,113) | 2.34 (0.89 to 3.83) | 62,634 (23,855 to 102,741) | 59.71 (22.68 to 97.94) | 0 (-0.12 to 0.12) | 0.17 (0.03 to 0.3) |
| Timor-Leste | 4 (1 to 6) | 1.40 (0.55 to 2.20) | 115 (44 to 186) | 34.54 (13.4 to 54.85) | 12 (5 to 20) | 1.53 (0.61 to 2.41) | 325 (124 to 515) | 36.66 (14.21 to 57.96) | 0.40 (0.24 to 0.57) | 0.28 (0.08 to 0.47) |
| Togo | 9 (4 to 14) | 0.86 (0.35 to 1.33) | 259 (105 to 398) | 19.70 (7.96 to 30.33) | 36 (15 to 61) | 1.1 (0.45 to 1.83) | 998 (400 to 1,701) | 24.53 (10.00 to 41.88) | 0.95 (0.88 to 1.02) | 0.84 (0.77 to 0.91) |
| Tokelau | 0 (0 to 0) | 2.05 (0.84 to 3.26) | 1 (0 to 1) | 48.61 (19.94 to 77.87) | 0 (0 to 0) | 1.84 (0.72 to 2.81) | 1 (0 to 1) | 43.79 (17.12 to 68.85) | -0.35 (-0.37 to -0.33) | -0.38 (-0.41 to -0.35) |
| Tonga | 1 (0 to 1) | 1.29 (0.53 to 1.96) | 17 (7 to 27) | 29.91 (12.12 to 46.69) | 1 (0 to 2) | 1.33 (0.53 to 2.05) | 24 (10 to 37) | 30.06 (11.92 to 46.06) | 0.12 (0.01 to 0.23) | -0.03 (-0.12 to 0.07) |
| Trinidad and Tobago | 23 (9 to 34) | 2.94 (1.21 to 4.45) | 545 (223 to 826) | 64.70 (26.56 to 98.18) | 52 (19 to 84) | 2.73 (1.01 to 4.37) | 1,272 (467 to 2,078) | 66.21 (24.33 to 108.1) | -0.21 (-0.34 to -0.09) | 0.03 (-0.10 to 0.15) |
| Tunisia | 68 (29 to 104) | 1.50 (0.64 to 2.32) | 1,726 (719 to 2,664) | 33.77 (14.22 to 52.11) | 181 (67 to 291) | 1.42 (0.53 to 2.28) | 4,420 (1,614 to 7,153) | 32.73 (12.01 to 52.74) | -0.34 (-0.41 to -0.26) | -0.26 (-0.32 to -0.19) |
| **Location** | **1990** | | | | **2021** | | | | **EAPC(1990-2021)** | |
|  | **Deaths Cases** | **ASMR**  **(/100k)** | **DALYs** | **ASDR**  **(/100k)** | **Deaths Cases** | **ASMR**  **(/100k)** | **DALYs** | **ASDR**  **(/100k)** | **ASMR** | **ASDR** |
|  | **No.**  **(95% UI)** | **No.**  **(95% UI)** | **No.**  **(95% UI)** | **No.**  **(95% UI)** | **No.**  **(95% UI)** | **No.**  **(95% UI)** | **No.**  **(95% UI)** | **No.**  **(95% UI)** | **No.**  **(95% CI)** | **No.**  **(95% CI)** |
| Turkmenistan | 23 (10 to 35) | 1.21 (0.51 to 1.83) | 690 (293 to 1,037) | 32.70 (13.87 to 49.16) | 39 (16 to 63) | 0.98 (0.39 to 1.59) | 1,164 (465 to 1,904) | 26.26 (10.49 to 42.86) | -0.65 (-1.14 to -0.16) | -0.69 (-1.17 to -0.21) |
| Tuvalu | 0 (0 to 0) | 2.13 (0.89 to 3.38) | 4 (2 to 6) | 52.53 (22.04 to 83.5) | 0 (0 to 0) | 2.01 (0.8 to 3.12) | 5 (2 to 8) | 48.43 (19.13 to 75.61) | -0.21 (-0.23 to -0.19) | -0.29 (-0.31 to -0.26) |
| Türkiye | 1,035 (430 to 1,555) | 3.16 (1.32 to 4.79) | 28,886 (12,195 to 44,150) | 77.50 (32.29 to 117.9) | 2,199 (932 to 3,389) | 2.44 (1.03 to 3.77) | 53,261 (22,828 to 83,118) | 56.2 (24.04 to 87.16) | -0.82 (-1.16 to -0.48) | -1.12 (-1.43 to -0.8) |
| Uganda | 104 (43 to 167) | 1.77 (0.74 to 2.82) | 2,829 (1,163 to 4,495) | 41.85 (17.33 to 66.47) | 246 (96 to 386) | 1.81 (0.70 to 2.85) | 7,037 (2,773 to 11,000) | 42.93 (16.73 to 67.46) | -0.42 (-0.64 to -0.19) | -0.49 (-0.74 to -0.24) |
| Ukraine | 2,770 (1,138 to 4,123) | 3.86 (1.59 to 5.75) | 70,707 (29,228 to 105,640) | 99.20 (40.9 to 148.1) | 2,323 (930 to 3,642) | 2.95 (1.18 to 4.63) | 56,144 (22,607 to 88,743) | 74.6 (30.11 to 117.8) | -1.09 (-1.23 to -0.95) | -1.23 (-1.39 to -1.08) |
| United Arab Emirates | 14 (5 to 23) | 3.31 (1.25 to 5.32) | 478 (172 to 784) | 79.73 (29.66 to 129.65) | 61 (24 to 107) | 2.45 (0.99 to 4.04) | 2,097 (818 to 3,721) | 49.15 (19.6 to 81.96) | 0.78 (0.23 to 1.33) | -0.18 (-0.62 to 0.26) |
| United Kingdom | 4,212 (1,795 to 6,264) | 4.53 (1.93 to 6.74) | 86,419 (36,523 to 128,269) | 99.13 (41.88 to 147.18) | 4,104 (1,761 to 6,211) | 2.91 (1.26 to 4.4) | 77,000 (32,919 to 116,035) | 62.02 (26.4 to 93.57) | -1.45 (-1.56 to -1.34) | -1.56 (-1.67 to -1.45) |
| **Location** | **1990** | | | | **2021** | | | | **EAPC(1990-2021)** | |
|  | **Deaths Cases** | **ASMR**  **(/100k)** | **DALYs** | **ASDR**  **(/100k)** | **Deaths Cases** | **ASMR**  **(/100k)** | **DALYs** | **ASDR**  **(/100k)** | **ASMR** | **ASDR** |
|  | **No.**  **(95% UI)** | **No.**  **(95% UI)** | **No.**  **(95% UI)** | **No.**  **(95% UI)** | **No.**  **(95% UI)** | **No.**  **(95% UI)** | **No.**  **(95% UI)** | **No.**  **(95% UI)** | **No.**  **(95% CI)** | **No.**  **(95% CI)** |
| United Republic of Tanzania | 130 (55 to 208) | 1.31 (0.57 to 2.07) | 3,631 (1,520 to 5,889) | 31.49 (13.31 to 50.51) | 351 (140 to 560) | 1.50 (0.61 to 2.37) | 9,584 (3,795 to 15,441) | 34.72 (13.75 to 55.48) | 0.46 (0.41 to 0.5) | 0.30 (0.25 to 0.36) |
| United States of America | 11,983 (5,096 to 17,838) | 3.67 (1.56 to 5.45) | 259,659 (109,797 to 383,680) | 83.29 (35.21 to 122.9) | 13,474 (5,747 to 20,241) | 2.29 (0.98 to 3.44) | 303,637 (129,374 to 454,027) | 56.53 (24.01 to 84.44) | -1.69 (-1.76 to -1.62) | -1.37 (-1.43 to -1.30) |
| United States Virgin Islands | 3 (1 to 5) | 3.95 (1.7 to 6.05) | 77 (32 to 116) | 88.35 (37.18 to 135.4) | 5 (2 to 7) | 2.55 (0.98 to 3.99) | 97 (37 to 154) | 59.62 (22.59 to 95.13) | -1.27 (-1.5 to -1.04) | -1.09 (-1.30 to -0.89) |
| Uruguay | 192 (80 to 288) | 4.91 (2.07 to 7.38) | 4,181 (1,740 to 6,285) | 109.38 (45.56 to 164.55) | 310 (125 to 462) | 5.18 (2.09 to 7.73) | 5,983 (2,424 to 8,918) | 112.34 (45.68 to 168.38) | 0.04 (-0.06 to 0.14) | -0.01 (-0.10 to 0.09) |
| Uzbekistan | 135 (54 to 209) | 1.17 (0.47 to 1.82) | 3,998 (1,602 to 6,101) | 32.47 (12.97 to 49.78) | 241 (93 to 379) | 0.91 (0.35 to 1.43) | 7,345 (2,861 to 11,567) | 24.62 (9.54 to 38.78) | -0.71 (-1.12 to -0.30) | -0.9 (-1.32 to -0.47) |
| Vanuatu | 1 (0 to 2) | 1.91 (0.74 to 3.10) | 32 (13 to 54) | 46.55 (18.25 to 75.96) | 3 (1 to 5) | 1.88 (0.75 to 3.01) | 89 (35 to 146) | 46.21 (18.33 to 74.16) | -0.11 (-0.17 to -0.04) | -0.12 (-0.22 to -0.03) |
| Venezuela (Bolivarian Republic of) | 138 (57 to 205) | 1.48 (0.61 to 2.20) | 3,706 (1,549 to 5,518) | 35.51 (14.83 to 52.99) | 496 (176 to 797) | 1.70 (0.60 to 2.74) | 12,727 (4,460 to 20,500) | 41.85 (14.67 to 67.29) | 0.40 (0.29 to 0.51) | 0.47 (0.36 to 0.59) |
| **Location** | **1990** | | | | **2021** | | | | **EAPC(1990-2021)** | |
|  | **Deaths Cases** | **ASMR**  **(/100k)** | **DALYs** | **ASDR**  **(/100k)** | **Deaths Cases** | **ASMR**  **(/100k)** | **DALYs** | **ASDR**  **(/100k)** | **ASMR** | **ASDR** |
|  | **No.**  **(95% UI)** | **No.**  **(95% UI)** | **No.**  **(95% UI)** | **No.**  **(95% UI)** | **No.**  **(95% UI)** | **No.**  **(95% UI)** | **No.**  **(95% UI)** | **No.**  **(95% UI)** | **No.**  **(95% CI)** | **No.**  **(95% CI)** |
| Viet Nam | 497 (202 to 767) | 1.26 (0.52 to 1.96) | 13,488 (5,386 to 20,889) | 32.36 (12.98 to 50.17) | 1,792 (693 to 2,823) | 1.86 (0.72 to 2.93) | 48,427 (18,721 to 77,348) | 45.98 (17.83 to 72.64) | 1.40 (1.28 to 1.52) | 1.33 (1.23 to 1.43) |
| Yemen | 59 (21 to 99) | 1.27 (0.48 to 2.10) | 1,745 (628 to 3,009) | 32.13 (11.72 to 54.31) | 175 (72 to 299) | 1.31 (0.54 to 2.21) | 5,102 (2,067 to 8,890) | 32.01 (13.14 to 55.15) | 0.16 (0.10 to 0.22) | 0.04 (-0.03 to 0.11) |
| Zambia | 39 (16 to 62) | 1.48 (0.61 to 2.32) | 1,136 (462 to 1,807) | 36.32 (14.97 to 57.54) | 139 (53 to 308) | 2.15 (0.85 to 4.29) | 4,206 (1,567 to 9,985) | 52.23 (19.77 to 115.88) | 1.22 (1.13 to 1.30) | 1.12 (1.03 to 1.22) |
| Zimbabwe | 516 (26 to 97) | 1.75 (0.74 to 2.74) | 1,638 (696 to 2,559) | 39.14 (16.71 to 61.04) | 144 (58 to 229) | 2.28 (0.92 to 3.59) | 4,270 (1,718 to 6,916) | 55.09 (21.93 to 87.67) | 1.21 (0.77 to 1.65) | 1.49 (0.97 to 2.01) |
